# Enhanced brain structure-function tethering in transmodal cortex revealed by high-frequency eigenmodes

**DOI:** 10.1038/s41467-023-42053-4

**Published:** 2023-10-24

**Authors:** Yaqian Yang, Zhiming Zheng, Longzhao Liu, Hongwei Zheng, Yi Zhen, Yi Zheng, Xin Wang, Shaoting Tang

**Affiliations:** 1 School of Mathematical Sciences, Beihang University, Beijing, 100191 China; 2 Key Laboratory of Mathematics, Informatics and Behavioral Semantics (LMIB), Beihang University, Beijing, 100191 China; 3 Institute of Artificial Intelligence, Beihang University, Beijing, 100191 China; 4 State Key Lab of Software Development Environment (NLSDE), Beihang University, Beijing, 100191 China; 5 Zhongguancun Laboratory, Beijing, China; 6 Beijing Advanced Innovation Center for Future Blockchain and Privacy Computing, Beihang University, Beijing, 100191 China; 7 PengCheng Laboratory, Shenzhen, 518055 China; 8 Institute of Medical Artificial Intelligence, Binzhou Medical University, Yantai, 264003 China; 9 School of Mathematical Sciences, Dalian University of Technology, Dalian, 116024 China; 10 Beijing Academy of Blockchain and Edge Computing (BABEC), Beijing, 100085 China

**Keywords:** Computational neuroscience, Cognitive neuroscience

## Abstract

While the link between brain structure and function remains an ongoing challenge, the prevailing hypothesis is that the structure-function relationship may itself be gradually decoupling from unimodal to transmodal cortex. However, this hypothesis is constrained by the underlying models which may neglect requisite information. Here we relate structural and functional connectivity derived from diffusion and functional MRI through orthogonal eigenmodes governing frequency-specific diffusion patterns. We find that low-frequency eigenmodes contribute little to functional interactions in transmodal cortex, resulting in divergent structure-function relationships. Conversely, high-frequency eigenmodes predominantly support neuronal coactivation patterns in these areas, inducing structure-function convergence along a unimodal-transmodal hierarchy. High-frequency information, although weak and scattered, could enhance the structure-function tethering, especially in transmodal association cortices. Our findings suggest that the structure-function decoupling may not be an intrinsic property of brain organization, but can be narrowed through multiplexed and regionally specialized spatiotemporal propagation regimes.

## Abstract (web summary)

Brain structure and function have been observed to be increasingly untethered in transmodal cortex. Here, the authors show that structure-function coupling in these areas can be enhanced by diffusion patterns governed by high-frequency eigenmodes.

## Introduction

The structural connectome shapes and constrains signaling transmission between neuronal populations, giving rise to complex neuronal coactivation patterns that are thought to support perception, cognition, and other mental functions^1^. Understanding the relationship between structure and function is a fundamental question in neuroscience^2^. With the development of network science and imaging techniques, numerous models have been proposed to investigate how the brain’s structural organization shapes its functional interaction patterns, including statistical models, communication models, and biophysical models^3–7^. A gradually emerging consensus is that functional connections can be inferred by collective, high-order interactions among neural elements, which transcends a simple one-to-one mapping between structural and functional connectivity^3,8^. Despite these modeling advances, the structure-function correspondence is relatively moderate, with structural connectivity rarely accounting for >50% of the variance in empirical functional connectivity^9^.

Recently, studies of regional structure-function relationships, using varying approaches such as communication models^9^, statistical correlation^10^, and graph harmonic analysis^11^, have independently found that the strength of structure-function coupling systematically varies across the brain. Structure and function are tightly coupled in primary sensorimotor areas but diverge in polysensory association areas, gradually decoupling along a macroscale functional gradient from unimodal to transmodal cortex^12^. Such heterogeneous structure-function correspondence raises the possibility that function cannot be completely predicted by structure alone, implying that the observed structure-function decoupling might be a natural consequence of brain hierarchical microscale organization, including cytoarchitecture^13^, intracortical myelination^14^, and laminar differentiation^15^. One prominent account posits that the rapid evolutionary expansion of the cortical mantle effectively releases association areas from early sensory-motor hierarchies, resulting in great signal variance and weak structure-function relationship in transmodal cortex^16^.

Though widely accepted, the corollary that the structure-function relationship may itself be decoupling in transmodal cortex seems to contradict reality. First, it has been widely believed that the brain network is organized under a trade-off between cost minimization and functionality maximization^17^. If structure contributed little to function, it would be unnecessary to invest such substantial material and metabolic resources in white matter construction in transmodal cortex^18–20^. Second, structure-function divergence confers considerable flexibility to structural connectome organization: any wiring diagram that guarantees the connection profile of unimodal cortex would be as good as any other in maintaining normal brain function. However, abundant empirical evidence indicates that structural connections exhibit a high level of consistency and specificity^21,22^. Third, the structure and function of association areas always change simultaneously. The development of human advanced cognitive capabilities was accompanied by pronounced modifications to connections linking association areas^23^ and abnormalities in these connections were reported to be associated with many neuropsychiatric disorders^24–26^. Such covariation implies a close correspondence between structure and function in polysensory transmodal areas.

If structure and function are indeed related, why do we observe the decoupling relationship in transmodal cortex? A possible explanation is that current models leave out requisite neurophysiological processes and signaling patterns when linking structural and functional connectivity. Neuromodulation and microstructural variations fundamentally influence how signals are routed, transformed, and integrated on the underlying anatomical backbone, ultimately manifesting as complicated functional connectivity patterns at the macroscale. Specifically, neuromodulation enables the static structural network to support distinct spatiotemporal propagation regimes^27^ while local attributes may elicit regional heterogeneity in signaling strategies and transmission events^28^. Nevertheless, most existing models only embody several putative neurophysiological mechanisms and tend to relate structure and function in the same way across the brain, inherently limiting the extent to which functional connectivity can be explained by structural connections. For instance, geometric and topological relationships (such as Euclidean distance, path length, and communicability) between nodes in the structural network are common and powerful predictors in functional connectivity reconstruction. However, these correlated predictors open the possibility of potentially homogeneous communication patterns, potentially inducing systematic deviation in structure-function coupling across the brain.

Hence, it remains a debate whether the structure-function divergence in association cortex is an inherent property of brain organization or a limitation of existing models. Although recent evidence from a machine learning approach demonstrates that structure-function prediction accuracies can be significantly improved^29^, it does not provide mechanistic insight into dynamical processes and activation patterns that underlie functional interactions. Whether structure and function are related in transmodal cortex, and if so, what mechanisms and dynamics govern this relationship remain exciting open questions. Here, we aim to shed light on these questions by assessing regional structure-function relationships using distinct frequency-specific spatiotemporal patterns generated by the eigenmode approach^30,31^. The role of eigenmodes as mediators of information transmission within the brain arises naturally from the network-diffusion model, where the eigenvalues closely relate to spatial complexity and persistent time of spreading processes^32–35^. Specifically, eigenmodes with near-zero eigenvalues, which are referred to as low-frequency eigenmodes, correspond to global and persistent spreading processes whereas eigenmodes with large eigenvalues, which are referred to as high-frequency eigenmodes, correspond to local and transient spreading processes. These orthogonal eigenmodes have attracted increasing attention in recent years^36–41^, opening new avenues to explore structure-function relationships. By predicting functional connectivity profiles of individual brain regions, we demonstrate that low-frequency eigenmodes, which are considered sufficient to capture the essentials of the whole-brain functional network, contribute little to functional connectivity in transmodal regions, leading to systematically decoupling relationships along the unimodal-transmodal gradient. In contrast, high-frequency eigenmodes, which are usually on the periphery of attention due to their association with noisy and random dynamical patterns, preferentially contribute to functional connectivity prediction in transmodal regions, inducing gradually convergent structure-function relationships from unimodal to transmodal regions. Although the information in high-frequency eigenmodes is weak and scattered, it could enhance the structure-function tethering, especially in transmodal cortex. These findings indicate that different brain regions may utilize specialized parallel spreading processes, that is, global, persistent diffusion patterns preferentially govern the structure-function tethering in unimodal cortex whereas local, transient dynamical processes play dominant roles in functional connectional profiles of transmodal cortex.

## Results

**Fig. 1:**
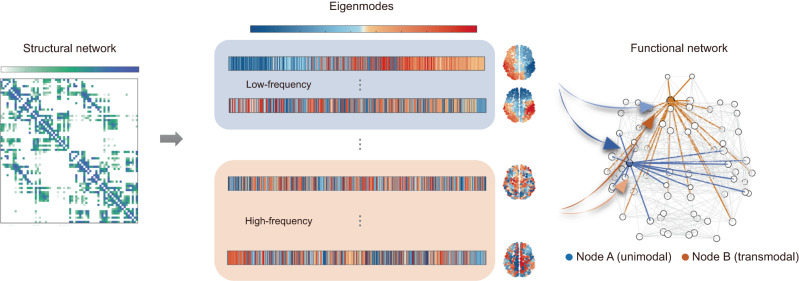
Method pipeline. Through the Laplacian eigendecomposition of the structural network, we obtained a series of orthogonal eigenmodes governing frequency-specific spatiotemporal patterns of signal propagation. The low-frequency and high-frequency components were respectively extracted to predict regional functional connection profiles via a multilinear regression model. The strength of structure-function coupling was measured as the Pearson correlation coefficient *R* between predicted and empirical connectivity profiles.

To explore how brain function coupled with structure through different diffusion processes, we applied the eigendecomposition to the structural connectome Laplacian and obtained a set of orthogonal eigenmodes governing frequency-specific spatiotemporal patterns of signal propagation (Fig. 1: left to middle panels). The low-frequency and high-frequency components of these eigenmodes were extracted to construct regional structure-function mappings via multilinear regression models (Fig. 1: middle to right panels). Specifically, for a given region, the dependent variable was the region’s functional connection profile, which represents the set of functional connectivity of that region to the rest of the brain. The predictor variables were low/high-frequency eigenmodes. The regional structure-function coupling was quantified as the goodness of fit, that is, the Pearson correlation (R) between the predicted and empirical functional connectivity profiles of brain regions. Our methodology followed the eigenmode approach with the difference that we focused on the regional structure-function relationships estimated by the extracted low-frequency and high-frequency components. Details of the analysis were provided in Methods.

The relationship between structural connectome organization and functional interaction patterns was explored through the structural and functional connectivity derived from diffusion and functional magnetic resonance imaging (See Methods). The dataset comes from the Lausanne University Hospital (LAU)^42^. Results were initially derived using group-consensus structural and functional connectivity networks parcellated at the highest resolution (1000nodes), and then replicated at the individual level, at another four resolutions (68, 114, 219, 448 nodes), as well as in an independently collected dataset (Human Connectome Project HCP)^43^.

### Regionally heterogeneous roles of Low-frequency eigenmodes

According to previous literature^36,44^, a small number of low-frequency eigenmodes are sufficient to capture the essence of the functional connectivity matrix. This observation, however, was made on whole-brain prediction where regional heterogeneity was neglected. It remains unclear what role low-frequency eigenmodes play in regional structure-function relationships.

**Fig. 2:**
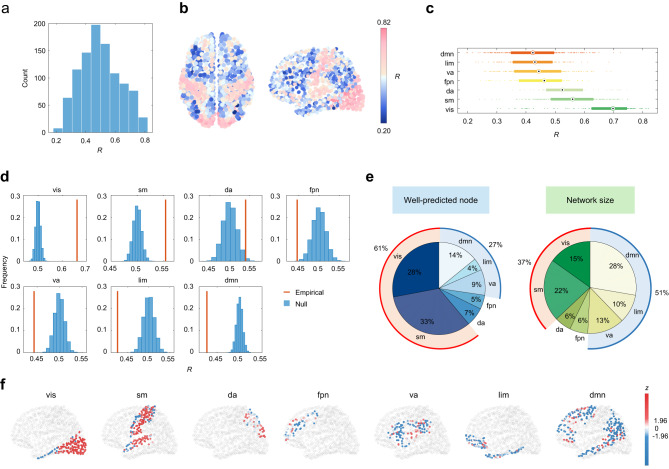
Heterogeneous contribution of low-frequency eigenmodes in regional structure-function prediction. Low-frequency eigenmodes, which are considered to be sufficient to capture the essence of the whole-brain functional network, are exploited to predict functional connection profiles of individual nodes. **a** The histogram of node-wise structure-function coupling *R* estimated by low-frequency eigenmodes. **b** The corresponding brain map where nodes are colored according to *R* values. **c** Node-wide *R* values (*n* = 1000 nodes) aggregated by 7 resting-state networks (RSNs): visual (vis), somatomotor (sm), dorsal attention (da), frontoparietal (fpn), ventral attention (va), limbic (lim), and default mode (dmn) networks. The boxplot shows the medians (circles), interquartile ranges (boxes), and min to max range (whiskers). **d** For each RSN, the network-specific mean *R* (red) was calculated and then compared with the null distribution (blue) generated by randomly permuting brain nodes’ assignments to seven resting-state networks (10,000 repetitions). **e** The distribution of well-predicted nodes whose *R* values are higher than the average level (left) and the distribution of network size across seven RSNs (right). **f** For each RSN, node-wise *R* values were transformed into z scores relative to the spatially-permuted null distribution. Source data are provided as a Source Data file.

To address this question, we estimated the regional structure-function relationships using a multilinear regression model that only comprise low-frequency eigenmodes (See Methods). The low-frequency eigenmodes were composed of the first ${K}_{L}$ components in increasing order of eigenvalues (here we set ${K}_{L}$ = 14 as a default value; the sensitivity analysis was subsequently performed for the robustness of results to threshold selection). The magnitude of structure-function coupling mirrors the contribution of low-frequency eigenmode to functional connectivity reconstruction. As shown in Fig. 2a, the distribution of regional coupling *R* was broad (from *R* = 0.2 to *R* = 0.8), suggesting highly variable roles of low-frequency eigenmodes in local structure-function prediction. We then examined the spatial distribution of regional coupling values (Fig. 2b). We found weak structure-function coupling in the bilateral inferior parietal lobule, lateral temporal cortex, precuneus, and inferior and middle frontal gyri. Conversely, strong structure-function coupling resided bilaterally in the visual and primary somatosensory cortices. Aggregating node-wise coupling values by seven resting-state networks proposed by Yeo et al. ^45^, we found structure and function were gradually decoupled from the unimodal cortex (visual and somatomotor networks) to the transmodal cortex (default mode network), suggesting that the contribution of low-frequency eigenmodes varies systematically across functional systems (Fig. 2c).

We further calculated the average *R* of each resting-state network and compared it with the null distribution generated by randomly permuting brain nodes’ assignments to seven resting-state networks (10,000 repetitions). The null hypothesis is that there exist no network-specific effects (or more technically, that network-specific average *R* is not different from those generated by random permutations). The P-value was calculated as the proportion of spatially-permutated network-specific *R* values that were more extreme than the observed network-specific R, and then was corrected for multiple comparisons. As illustrated in Fig. 2d, we found that the structure-function coupling in visual and somatomotor networks was stronger than the null distribution while ventral attention, limbic, and default mode networks exhibited weaker structure-function coupling than the level expected by chance. Although these differences were overall modest in magnitude, they were statistically significant (FDR corrected *P* < 10^−4^), implying that the observed heterogeneous contribution of low-frequency eigenmodes is not a trivial pattern but is potentially circumscribed by functional systems. We also compared the distribution of well-predicted nodes whose prediction accuracies were higher than the average level with the distribution of seven resting-state network sizes (Fig. 2e). If low-frequency eigenmodes contribute equally to the structure-function coupling across brain regions, these two distributions would exhibit strong similarity. However, we observed an apparent discrepancy between the distributions. We found that 61% of well-predicted nodes were concentrated in visual and somatomotor networks, which greatly exceeded the corresponding network size (37%). In contrast, 27% of well-predicted nodes were observed in default mode, limbic, and ventral attention networks, which was much smaller than the corresponding network size (51%). Finally, we transformed the node-wise *R* to its *z* score concerning the null distribution of each resting-state network. Positive values indicate that structure and function are tightly linked by low-frequency eigenmodes whereas negative values indicate that low-frequency eigenmodes play a poor role in predicting functional connectivity. As shown in Fig. 2f, we observed a gradually worsening performance of low-frequency eigenmodes in relating structure to function from unimodal to transmodal cortex.

Collectively, these findings suggest that the contribution of low-frequency eigenmodes to structure-function prediction is not uniform across the brain. The persistent, spatially slow-varying diffusion patterns captured by low-frequency eigenmodes can adequately explain functional connection profiles of unimodal regions. However, they contribute little to the functional connectivity of transmodal regions, leading to the observed structure-function divergence in these regions.

### Structure-function decoupling induced by low-frequency eigenmodes

Besides the spatially heterogeneous contribution of low-frequency eigenmodes, there exist many other systematic variations^46^ in brain organization. Margulies et al.^12^ reported a cortical organization from unimodal to transmodal cortex, which simultaneously corresponds to a spectrum of increasingly abstract cognitive functions. Here, we associated the regional structure-function relationship estimated by low-frequency eigenmodes with this macroscale functional gradient to examine whether coupling heterogeneities vary along the unimodal-transmodal hierarchy.

**Fig. 3:**
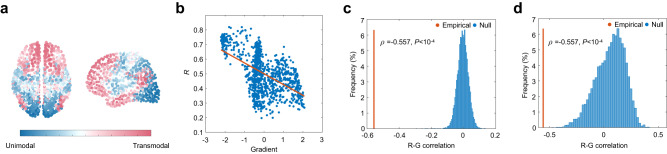
Structure-function decoupling along the unimodal-transmodal hierarchy. **a** The macroscale functional gradient spanning from unimodal to transmodal cortex. **b** Structure-function coupling *R* estimated by low-frequency eigenmodes is negatively correlated with the macroscale functional gradient. **c** Red: The empirical Pearson correlation coefficient *ρ* between coupling *R* and the functional gradient. Blue: the null distribution of correlation coefficients generated by randomly rotating nodes’ spatial positions (10,000 repetitions). **d** Red: correlation coefficient *ρ* of empirical data. Blue: the null distribution generated by randomizing structural architecture (10,000 repetitions). **c**, **d** We provided the empirical *P* values (one-sided, unadjusted), calculated as the proportion of correlation coefficients generated by the null model that were lower than the observed correlation coefficients. Source data are provided as a Source Data file.

We derived the functional gradient following the method in ref. ^12^ and correlated it with the node-wise coupling values. Briefly, we applied diffusion map embedding^47^, a nonlinear dimensionality reduction approach, to the functional connectivity matrix, generating a transition matrix that was subjected to eigendecomposition. The first eigenvector of this matrix was what we referred to as a macroscale functional gradient, characterizing a hierarchical organization that situated unimodal and transmodal cortex on opposite extremes (Fig. 3a). As shown in Fig. 3b, we found a negative correlation between structure-function coupling *R* and the functional gradient (Pearson *ρ* = −0.557). To examine whether such anticorrelation is a meaningful feature of the empirical structural connectome, we generated two types of null models. The first one kept the structural connection topology fixed but randomly rotated nodes’ spatial positions^48^. The second one preserved the original spatial embedding but incorporated no structural information except the degree sequence. As shown in Fig. 3c, d, the correlation coefficient between the coupling *R* and the functional gradient in the empirical data was significantly lower than those generated by the two null models (*P* < 10^−4^, 10,000 simulations). This observation suggests that structure-function decoupling from unimodal to transmodal cortex is a nontrivial pattern induced by low-frequency eigenmodes. More specifically, if we link brain structure and function only through spreading processes sustained by low-frequency eigenmodes, the resulting structure-function relationships will become increasingly divergent along the unimodal-transmodal hierarchy.

### Information in high-frequency eigenmodes

Although whole-brain functional connectivity can be efficiently captured by a few low-frequency eigenmodes, the correspondence between structure and function is far from perfect. It remains a matter of debate whether structure-function divergence in transmodal regions is an inherent property of brain organization or a consequence of neglecting information requisite for precise prediction.

To test the latter possibility, we first examined whether eigenmodes apart from low-frequency components made significant contributions to functional brain connectivity. We applied each eigenmode to whole-brain structure-function prediction and transformed the prediction accuracy *R* into a z score relative to the null distribution generated by the corresponding pseudo-eigenmode. The pseudo-eigenmodes are the phase-randomization of empirical eigenmodes while preserving the original spatial frequency, and have been recently applied to build null benchmarks in^49–51^. We found that a large number of eigenmodes with large eigenvalues significantly outperform the corresponding pseudo-eigenmodes in structure-function prediction, although the prediction accuracy decreased steeply from low-frequency to high-frequency domains (Supplementary Fig. S1). This observation suggests that high-frequency eigenmodes may contain weak but valuable information for structure-function coupling.

**Fig. 4:**
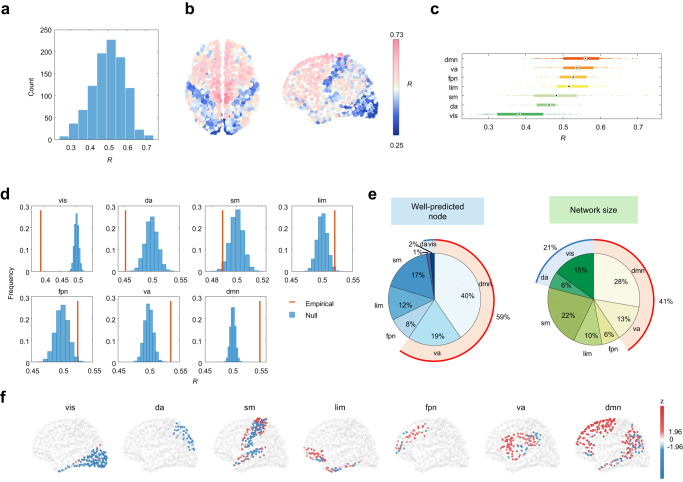
The regional prediction contribution of high-frequency eigenmodes. **a** A wide distribution of node-wise Pearson correlation *R* across 1000 nodes. **b** The corresponding brain map where nodes are colored from blue to red in increasing order of *R* values. **c** Node-wide *R* values (*n* = 1000 nodes) aggregated by 7 resting-state networks (RSNs). The boxplot shows the medians (circles), interquartile ranges (boxes), and min to max range (whiskers). **d** The average *R* value of each RSN is compared with those calculated after random permutation of nodes’ assignments (10,000 repetitions). **e** The distribution of well-predicted nodes across seven RSNs is compared with the distribution of network size. **f** The spatial distribution of z scores within each RSN. Source data are provided as a Source Data file.

We then investigated how regional functional connectivity emerges from the underlying structure substrate through transient, geometrically complex diffusion patterns captured by high-frequency eigenmodes. The high-frequency eigenmodes were selected in descending order of eigenvalues (here we set ${K}_{H}=434$ as a default value; the sensitivity analysis was subsequently performed for the robustness of results to threshold selection). We used a multilinear regression model with high-frequency eigenmodes as predictors for regional prediction (See Methods). The magnitude of the structure-function coupling *R* reflects the contribution of high-frequency eigenmodes. In Fig. 4a, we showed that the *R* values varied from 0.25 to 0.73, suggesting heterogeneous roles of high-frequency eigenmodes across the brain cortex. Furthermore, the spatial distribution of *R* values indicates a systematic variation in the strength of structure-function coupling (Fig. 4b). Structure and function are closely aligned in prefrontal and paracentral cortices but decoupled in visual and primary somatosensory cortices, exhibiting a coupling pattern inverse to that induced by the low-frequency eigenmodes. Aggregating accuracy *R* by seven functional systems, we found the default mode network exhibited the strongest structure-function coupling while the visual network exhibited the weakest structure-function coupling (Fig. 4c).

Similarly, to examine whether the regionally heterogeneous contribution of high-frequency eigenmodes is system-specific, we compared the average *R* of each functional network with the null distribution generated by randomizing nodes’ assignments (10,000 repetitions). As shown in Fig. 4d, we found that the structure-function coupling in ventral attention and default mode networks was higher than the null distribution while the dorsal attention and visual networks exhibited weaker structure-function coupling than the level expected by chance. These differences were overall modest but statistically significant (FDR corrected *P* < 10^−4^), implying that the observed heterogeneous contribution of high-frequency eigenmodes is a nontrivial pattern determined by the partition of functional systems. We further compared the distribution of well-predicted nodes with the distribution of functional network size to rule out the influence of differences in network size. We found that the proportion of well-predicted nodes in ventral attention and default mode networks was much higher than the corresponding network size (59% >41%) whereas the reverse was true for the dorsal attention and visual networks (3% <21%) (Fig. 4e). Mapping coupling *z* scores back to individual brain regions, we found that strong structure-function correspondence was concentrated in transmodal regions (Fig. 4f). This observation suggests that the diffusion patterns captured by high-frequency eigenmodes preferentially contribute to the interpretation of functional interactions in transmodal regions.

### Structure-function convergence in transmodal cortex induced by high-frequency eigenmodes

**Fig. 5:**
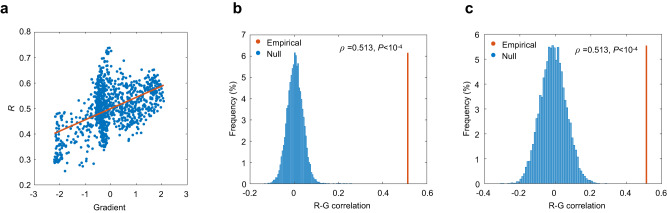
Convergent structure-function relationships along the unimodal-transmodal gradient. **a** Structure-function coupling *R* estimated by high-frequency eigenmodes is positively correlated with the functional gradient. **b** The empirical correlation coefficient (red line) is significantly larger than the null distribution generated by randomly rotating nodes’ spatial positions (10,000 repetitions; blue). **c** The empirical correlation coefficient (red line) is significantly larger than those obtained from artificial structural networks (10,000 repetitions; blue). **b**, **c** We provided the empirical *P* values (one-sided, unadjusted), calculated as the proportion of correlation coefficients generated by the null model that were greater than the observed correlation coefficients. Source data are provided as a Source Data file.

To investigate how local structure-function relationships estimated by high-frequency eigenmodes vary along the unimodal-transmodal hierarchy, we associated the regional coupling pattern with the macroscale functional gradient. As shown in Fig. 5a, we found these two measures were positively correlated (Pearson *ρ* = 0.513), suggesting that structural and functional connectivity are increasingly coupled from unimodal to transmodal regions under transient and spatially complex diffusion processes captured by high-frequency eigenmodes. To assess the significance of this spatial correlation, we compared the empirical correlation coefficient with a null distribution generated by spatial permutation with spatial autocorrelation preserved (Fig. 5b). We found that the empirical correlation coefficient was significantly larger than the null values ($P < {10}^{-4}$, 10,000 simulations). We also constructed a null model by rewiring network edges while preserving the degree sequence, which disrupts the network topology (Fig. 5c). As one might expect, the empirical correlation coefficient was significantly higher than those generated by the null model ($P < {10}^{-4}$, 10,000 simulations), suggesting that such increasing convergence between structure and function from unimodal to transmodal cortex is a nontrivial pattern of the empirical connectome.

### Regional structure-function coupling at the individual level

To improve the signal-to-noise ratio, we initially performed our analysis on group-average structural and functional networks. In this section, we sought to understand the regional patterns of structure-function coupling estimated by low-frequency and high-frequency eigenmodes from the perspective of individual subjects.

**Fig. 6:**
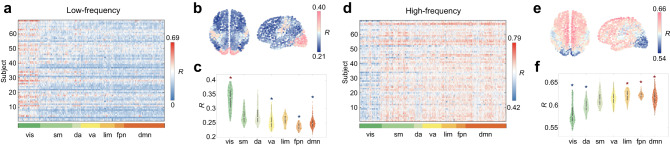
Spatial patterns at the individual level. **a** Regional structure-function coupling estimated by low-frequency eigenmodes for every region and subject. **b** The corresponding brain map where nodes are colored from blue to red in increasing order of *R* values. **c** Regional structure-function coupling grouped by 7 brain functional systems. Each point represents a brain region (*n* = 1000 regions). Boxplots are contained within violinplots, where the white circle denotes the median, the box denotes the interquartile range, and the whiskers denote the upper/lower bounds of 1.5 $\times$ the interquartile range. The network-specific mean *R* was then compared with the null distribution generated by spatially constrained permutation (10,000 repetitions). * indicates the empirical *P* < 10^−4^ (two-sided, FDR corrected). **d** Regional structure-function coupling estimated by high-frequency eigenmodes for every region and subject. **e** The corresponding brain map where nodes are colored from blue to red in increasing order of *R* values. **f** Regional structure-function coupling grouped by 7 brain functional systems. Each point represents a brain region (*n* = 1000 regions). Boxplots are contained within violinplots, where the white circle denotes the median, the box denotes the interquartile range, and the whiskers denote the upper/lower bounds of 1.5 $\times$ the interquartile range. The network-specific mean *R* was then compared with the null distribution generated by spatially constrained permutation (10,000 repetitions). * indicates the empirical *P* < 10^−4^ (two-sided, FDR corrected). Source data are provided as a Source Data file.

For low-frequency eigenmodes, we conducted the fitting procedure for every subject, which returns a matrix of coupling *R* whose size is [69 subjects × 1000 region] (fMRI data were missing for one participant; Fig. 6a). As in previous sections, we found considerable variability across regions (one-way ANOVA R; F(999) = 11.9; *P* < 10^−15^), confirming the regionally heterogeneous roles of low-frequency eigenmodes in local structure-function prediction. To visualize the spatial distribution of these results, we averaged over subjects and plotted the mean structure-function coupling *R* for each region (Fig. 6b). We found that the magnitude of structure-function coupling varied systematically across the cortex, with primary unimodal cortices exhibiting the higher prediction accuracies than transmodal association cortices. To assess whether the contributions of low-frequency eigenmodes were concentrated within specific functional systems, we aggregated these *R* values by seven functional networks and compared the network-specific mean *R* with the null distribution generated by a spatially-constrained permutation model (spin test; 10,000 permutations). As illustrated in Fig. 6c, we found that the visual network had significantly higher structure-function coupling relative to the null distribution while ventral attention, frontoparietal, and default mode networks exhibited lower *R* values than the level expected by chance (FDR corrected *P* < 10^−4^). Considering the inter-individual heterogeneity, we also provided the distributions of network-specific mean *R* over all subjects (Supplementary Fig. S2a) and repeated the analysis for every subject. The network-specific effects were significant at a single subject level (See Supplemental section “Analysis of individual subjects” for more details). We further correlated the regional coupling *R* with the unimodal-transmodal functional gradient for each subject, comparing the empirical correlation coefficients against those obtained using a spatially-constrained permutation model (1000 permutations) and against those generated by randomly rewiring network edges with degree sequence preserved (1000 permutations). We found that the correlation between the regional *R* and functional gradient was overall negative although these correlation coefficients were considerably variable across subjects (Pearson *ρ* = −0.297 ± 0.168; Supplementary Fig. S2b and Fig. S3).

For high-frequency eigenmodes, the structure-function coupling estimated at the individual level yielded a matrix containing *R* of every region and subject, which was reported in Fig. 6d. Consistent with the previous section, we observed regionally heterogeneous contributions of high-frequency eigenmodes to structure-function prediction (one-way ANOVA R; F(999) = 15.7; *P* < 10^−15^). Averaging regional *R* across subjects, we observed relatively weak structure-function coupling in unimodal primary and particularly in visual regions, and relatively strong coupling in transmodal association regions (Fig. 6e). To examine whether the contributions of high-frequency eigenmodes are system-specific, we aggregated these *R* values by seven functional networks, comparing the network-specific mean *R* with those obtained by the spatially-constrained permutation model (spin test; 10,000 permutations). As illustrated in Fig. 6f, we found that regions with lower *R* values were prominent in the visual and dorsal attention networks whereas regions with higher *R* values were affiliated with the limbic, frontoparietal, and default mode networks (FDR corrected *P* < 10^−4^). We also took into account inter-individual heterogeneity and plotted the distribution of network-specific mean *R* over all subjects (Supplementary Fig. S2c). Compared to the null distributions generated using a subject-specific spatially constrained permutation model, the observed network-specific effects were still statistically significant (See Supplemental section “Analysis of individual subjects” for more details). Furthermore, we found that the regional coupling *R* and functional gradient were overall positively correlated at a single individual level (Pearson *ρ* = 0.254 ± 0.157; Supplementary Fig. S2d and Fig. S4).

We further estimated the standard deviation of coupling *R* estimated by low-frequency and high-frequency eigenmodes for every region across all subjects (Supplementary Fig. S5). Interestingly, we found that regions with great inter-individual variations were overall concentrated in the visual and somatosensory cortex whereas structure-function coupling in prefrontal, lateral temporal, and inferior parietal cortex was relatively consistent across subjects. Note that, for low-frequency eigenmodes, this spatial pattern is very similar to the spatial distribution of regional coupling *R* (Pearson *ρ* = 0.79), and when transforming the standard deviation to the coefficient of variation, this trend did not persist (Pearson *ρ* = 0.05; *P* = 0.09), suggesting that the regional heterogeneity in inter-individual variability observed in the case of low-frequency eigenmodes may be attributable to a floor effect. In contrast, for high-frequency eigenmodes, the negative association between inter-individual variation in structure-function coupling and the unimodal-transmodal functional gradient was still statistically significant (Pearson *ρ* = −0.53; *P* < 10^−5^).

Collectively, these results suggest that the contribution of low-frequency eigenmodes is not uniform across the brain but concentrated on the primary unimodal regions, resulting in structure-function decoupling along the unimodal-transmodal gradient. Conversely, high-frequency eigenmodes preferentially contributed to the interpretation of functional profiles in transmodal association regions, inducing gradually convergent structure-function relationships from unimodal to transmodal regions. Both of these results are consistent with those obtained from group-average data. Furthermore, comparable spatial patterns of structure-function coupling were also with under different low/high-frequency thresholds (Supplementary Fig. S6), spatial resolutions (Supplementary Fig. S7), data acquisition (HCP; Supplementary Fig. S8), as well as the method of functional connectivity reconstruction (partial correlation; Supplementary Fig. S9). See Supplemental section “Sensitivity analyses” for more details.

### Enhanced structure-function tethering via introducing high-frequency eigenmodes

As a final step, we sought to shed light on two questions: Can high-frequency eigenmodes compensate for the critical information neglected in previous structure-function predictions? If so, how is this information distributed in high-frequency eigenmodes?

**Fig. 7:**
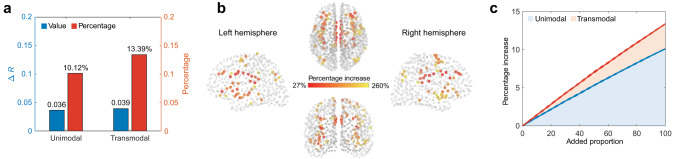
Enhanced structure-function tethering by high-frequency eigenmodes. **a** The increments (blue) and increase percentages (red) of prediction accuracy with the addition of high-frequency eigenmodes for unimodal and transmodal regions. **b** The spatial distribution of the top 10% of nodes with the highest percentage increases in prediction accuracy. **c** Growth curves of prediction accuracy for unimodal and transmodal groups. The horizontal axis indicates the proportion of the high-frequency eigenmodes added to the prediction model while the vertical axis is the percentage increase in *R*. At each adding step, the high-frequency eigenmode is randomly selected (10 repetitions). The average percentage increase is shown in solid lines while standard deviations are expressed as dash lines. Source data are provided as a Source Data file.

Firstly, we focused on the structure-function coupling estimated by prediction models with and without high-frequency eigenmodes. Considering many high-frequency eigenmodes, we used LASSO regression, which generates a sparse prediction model by penalizing regression coefficients to reduce the risk of overfitting. Briefly, for each brain region, we employed nested parameter optimization to tune the regularization parameters (which is strictly limited to the training set), and evaluated the performance by Pearson correlation between predicted and empirical functional connectivity profiles in the remaining test set. The brain regions were divided into two groups based on whether their function gradient values were larger than zero, yielding a unimodal group of 590 and a transmodal group of 410. We found that the prediction accuracy in both unimodal and transmodal groups improved with the addition of high-frequency eigenmodes (two-sided paired t-test, *P* < 10^−4^; Fig. 7a), suggesting that high-frequency eigenmodes provide supplementary information for structure-function tethering. We also found that the prediction improvement in the transmodal group was overall greater than that in the unimodal group and that the top 10% of brain regions with the highest percentage increases were mostly located in the inferior parietal cortex, insula, cingulate, and prefrontal cortex (Fig. 7b). These results indicate that the information in high-frequency eigenmodes is biased to functional connections in transmodal regions.

Further, to address the second question, we quantified the percentage increases in prediction accuracy along with the progressive addition of high-frequency eigenmodes. The high-frequency eigenmode added at each step was randomly selected and the adding process was repeated 10 times. The mean and the standard deviation of the percentage increase in *R* were illustrated in Fig. 7c. We found that the prediction accuracy in both unimodal and transmodal groups increased steadily as high-frequency eigenmodes were added gradually, suggesting that the information requisite for structure-function prediction is uniformly distributed across high-frequency eigenmodes. It is noteworthy that the growth curve of the transmodal group is steeper than that of the unimodal group, which consolidates the preference of high-frequency eigenmode for interpreting functional interaction in transmodal regions.

## Discussion

The imperfect correspondence between structure and function in macroscale brain networks is an ongoing challenge in network neuroscience^2^. The prevailing hypothesis is that structure and function may be gradually untethered along a macroscale functional gradient spanning from unimodal areas to transmodal areas^3^. In this work, we revisit this hypothesis on the grounds that typically prediction models may neglect signal propagation patterns that are critical for functional interactions in transmodal cortex. To gain a deeper understanding of how functional connectivity emerges from the underlying anatomical substrate, we take into account distinct networked diffusion processes by decomposing the structure connectome into frequency-specific diffusion patterns captured by orthogonal eigenmodes^30,31^. Concordant with previous findings^9–11^, a gradual decoupling between structure and function along unimodal-transmodal hierarchy is reproduced based on low-frequency eigenmodes which are reported as prominent predictors of whole-brain functional connectivity. Next, we show that apart from low-frequency eigenmodes, high-frequency eigenmodes also significantly contribute to structure-function prediction, even though the information they contain is weak and scattered. Unexpectedly, these high-frequency eigenmodes reverse the decoupling pattern between structure and function across the brain, inducing increasingly convergent structure-function relationships along the unimodal-transmodal hierarchy. Finally, we show that high-frequency eigenmodes could enhance the strength of structure-function coupling, especially in transmodal association cortex.

Our work contributes to understanding the link between structural and functional connectivity from a parallel communication perspective. The structure-function relationship has been fruitfully investigated by formulating models of potential communication dynamics, ranging from centralized forms such as the shortest path to decentralized forms such as the random walk^52^. However, the correlation of typical predictors such as path length^5^, navigation^53^, and communicability^54^ mirrors the homogeneity of potential signal propagation patterns which may drive systematic deviations in structure-function alignment. A key challenge lies in aggregating heterogeneous communication dynamics in a simple and unified framework and articulating their roles in functional interactions among neuronal elements. In our work, a variety of possible diffusion processes (orthogonal eigenmodes) were gleaned from the eigendecomposition of the structural Laplacian, with distinct eigenvalues reflecting different frequencies of spatiotemporal patterns of spreading processes^34,55^. Interregional functional interactions can be interpreted by activating these frequency-specific networked persistent modes in appropriate proportion. This methodology is in line with recent biophysical models which suggest the coexistence of a set of self-sustained, stimulus-selective activity states, with each one storing a memory item for optimal preparation for stimulus processing^56,57^. Studies investigating temporal dynamics of interregional synchrony also suggest that frequency-specific interactions, which form transient frequency-specific networks, modulate cortical computations and information transformation in the brain^58,59^.

For the present analysis, we focus on low-frequency and high-frequency eigenmodes that cover two fundamentally different types of diffusion patterns, one sustaining persistent and widespread spreading processes while the other capturing faster and more geometrically complex spreading processes^32,55^. A rich literature supports the notion that brain activity is preferentially expressed in low frequencies^11^ and argues that a small number of low-frequency eigenmodes are sufficient to reconstruct the functional network^34,36^. However, this perspective was largely based on the whole-brain prediction where structure-function relationships are assumed to be uniform across the cortex. We show that low-frequency eigenmodes contribute little to structure-function prediction in transmodal areas. In contrast, high-frequency eigenmodes, which are typically associated with noisy and random activation patterns^60,61^, promote the interpretation of functional connectional profiles in these areas. These findings advance the understanding of the roles of different-frequency eigenmodes in structure-function prediction, emphasizing the importance of high-frequency eigenmodes that used to be on the periphery of attention in eigenmode analyses. The significant contribution of low-frequency and high-frequency patterns highlights multiplexed strategies and multiple mechanisms involved in interregional communication^62–64^, suggesting that synchrony among neuronal populations may result from the aggregation of global, persistent, and local, transient spatiotemporal patterns. Physiological signals from distributed brain regions compete and cooperate in different frequency bands, manifesting as distinct synchronization patterns to serve flexible cognitive behaviors^65–67^. These various and abundant frequency-specific patterns allow neuronal elements to share and transmit information through dynamical reconfiguration on multiple timescales, potentially relaxing the restriction on the material and energy cost of the structural connectome^52,68^. Our findings gain valuable insight into how flexible flow of information is achieved, opening the possibility to address the major unsolved question that how static structural connectome supports fast and flexible reconfiguration of functional networks^51^.

Furthermore, our findings suggest regionally heterogeneous contribution of different-frequency eigenmodes in predicting functional interactions. Persistent and global diffusion patterns described by low-frequency eigenmodes predominantly explain functional connectional profiles of primary unimodal regions. Transient and geometrically complex diffusion processes captured by high-frequency eigenmodes instead support functional interactions in transmodal association cortex. These systematic variations in the prediction performance of different diffusion patterns may be induced by latent microstructural configurations and hierarchical organizing principles in the brain, including morphometric similarity, transcription profiles, and laminar differentiation^14,15,28^. The organization of primary areas is strongly constrained by molecular gradients and early activity cascades^69^. Information is step-wise progressively transformed along serial and hierarchical pathways^70^. Such consistent hierarchical property of unimodal cortex may thus elicit widespread and persistent spreading processes that can be captured by low-frequency eigenmodes. In contrast, the rapid expansion of the cerebral cortex detaches polysensory association areas from the canonical sensory-motor hierarchy, resulting in noncanonical circuit organization that lacks consistent laminar projection patterns^16,71^. Such variation in connectivity patterns may alter the way signals are generated, transformed, and integrated, potentially eliciting fundamentally different propagation patterns in association areas^72,73^. The association cortex is configured to bridge widely distributed functional systems and integrate diverse information from multiple sources^74–76^. Transient and geometrically complex spreading processes captured by high-frequency eigenmodes may enable transmodal cortex to participate in different communication events in a spatially and temporally precise manner, facilitating efficient information routing and flexible state switching in cognitive behaviors. Our findings are also corroborated by the previous work which suggests that primary sensory and motor networks are closely associated with low-frequency connectome harmonics while higher-order cognitive networks match a broader range of frequency spectrum^55^.

Low-frequency and high-frequency eigenmodes respectively induce gradually divergent and convergent structure-function relationships along the unimodal-transmodal gradient. These two reverse coupling patterns offer an alternative perspective for understanding the link between structure and function, that is, structural and functional connectivity may be tightly tethered but current models neglect requisite communication dynamics for precise prediction. With the assistance of high-frequency eigenmodes, the tethering between structure and function is enhanced in both unimodal regions and transmodal regions. In particular, the 10% highest increases in the strength of structure-function coupling appear in the inferior parietal cortex, insula, cingulate, and prefrontal cortex, suggesting that structure-function divergence in transmodal areas may be narrowed by additional information. This is in accordance with the recent study^29^ which exploits a machine learning approach to achieve a substantially closer structure-function correspondence than previously implied. Although the information in high-frequency eigenmodes is prone to be obscured by background noise and the exact mechanism underlying structure-function association requires further exploration, our findings open an opportunity to improve the understanding of structure-function tethering. Considering the steady and continuous growth of prediction accuracy with the increasing number of added high-frequency eigenmodes, the information is expected to be uniformly distributed in the high-frequency domain. Meanwhile, the growth curve of transmodal areas is steeper than that of unimodal areas, suggesting that high-frequency patterns have a propensity to explain neuronal coactivation in transmodal cortex. These results provide references for future work on distilling information from high-frequency eigenmodes to adequately capture the structure-function relationship.

There are some limitations to our work. First, although we have demonstrated the role of high-frequency eigenmodes in enhancing structure-function tethering, it is still difficult to extract useful information quickly and accurately due to the large amount of noise in high frequencies. Meanwhile, recent studies have demonstrated that regional structure-function alignments vary with individual differences (e.g., age, gender, and cognitive traits)^77–81^. Understanding how these individual differences affect regional structure-function coupling estimated by different diffusion processes is an exciting issue that requires further investigation, with potential implications for the diagnosis and treatment of brain disorders. Moreover, we represent functional interactions among neuronal elements simply as static and dyadic connectivity networks, neglecting the possibility of temporal dynamics^82,83^ and high-order interactions^84^. Future work could investigate structure-function coupling with more nuanced models enriched with dynamic and high-order interactions.

## Methods

### Data acquisition

The analyses were performed in two independent datasets. The main dataset was collected by Department of Radiology, University Hospital Center and University of Lausanne (LAU)^42^. The dataset was collected from a cohort of 70 healthy participants (27 females, 28.8$\pm$9.1 years old). Informed content approved by the Ethics Committee of Clinical Research of the Faculty of Biology and Medicine, University of Lausanne was obtained from all participants. Diffusion spectrum images (DSI) were acquired on a 3-Tesla MRI scanner (Trio, Siemens Medical, Germany) using a 32-channel head-coil. The protocol was comprised of (1) a magnetization-prepared rapid acquisition gradient echo (MPRAGE) sequence sensitive to white/gray matter contrast (1-mm in-plane resolution, 1.2-mm slice thickness), (2) a DSI sequence (128 diffusion-weighted volumes and a single b0 volume, maximum b-value 8000 s/mm^2^, 2.2$\times$2.2$\times$3.0 mm voxel size), and (3) a gradient echo EPI sequence sensitive to blood oxygen level-dependent (BOLD) contrast (3.3-mm in-plane resolution and slice thickness with a 0.3-mm gap, TR 1920 ms, resulting in 280 images per participant). The supplementary analyses were performed in the dataset from the Human Connectome Project (HCP)^43^. This dataset consisted of 56 participants. Informed content, including consent to share de-identified data, approved by the Washington University institutional review board was obtained from all participants. For more details regarding acquisitions see ref. ^85^.

### Structural and functional network reconstruction

For LAU, the initial signal processing of MPRAGE, DSI, and fMRI BOLD data was performed using the Connectome Mapper pipeline^86^. Gray and white matter were segmented from the MPRAGE volume and divided into 68 brain regions following Desikan–Killiany atlas^87^. These regions were further subdivided into 114, 219, 448, and 1000 approximately equally sized nodes according to the Lausanne anatomical atlas using the method proposed by^88^. DSI data were reconstructed following the protocol described by^89^. Structural connectivity matrices for individual participants were estimated using deterministic streamline tractography on reconstructed DSI data, initiating 32 streamline propagations per diffusion direction for each white matter voxel^90^. The measure of structural connectivity between pairs of regions was fiber density, defined as the normalized number of streamlines between two brain regions^91^. A group-average structural connectivity matrix was estimated using a consensus approach preserving the density and the edge-length distribution of the individual participant matrices^92–94^. Functional networks were reconstructed using fMRI data from the same individuals. fMRI volumes were corrected for physiological variables, including regression of white matter, cerebrospinal fluid, and motion. fMRI time series were lowpass filtered (temporal Gaussian filter with full-width half maximum equal to 1.92 s). The first four volumes were discarded and motion “scrubbing” was performed^95^. fMRI data were parcellated according to the same atlas used for structural networks. Functional connectivity matrices for individual participants were constructed by estimating the Pearson correlation between the fMRI time series of each pair of brain regions. Note that fMRI data were missing for one individual and we obtained functional connectivity matrices for 69 participants. A group-average functional connectivity matrix was constructed by: (1) concatenating the regional fMRI time series from all participants, (2) estimating the Pearson correlation between each pair of brain regions, and (3) thresholding. In the thresholding operation, 276 points from the concatenated times series were randomly sampled to estimate a correlation matrix. This process was repeated 1000 times, generating 1000 bootstrapped matrices. From these bootstrapped samples, confidence intervals for the correlation magnitude were estimated for each pair of brain regions. In the group-average functional connectivity matrix, only the functional connectivity between pairs of brain regions whose correlations were consistently positive or negative was retained, and functional connectivity between the remaining pairs of brain regions was set to zero.

For HCP, all acquisitions were preprocessed according to HCP-minimal preprocessing guidelines^85^. Glasser’s multimodal parcellation^96^ was used to divide the cortex into 360 regions. Individual structural networks were derived from diffusion-weighted imaging using the MRtrix3 package [http://www.mrtrix.org/]. The operations included multi-shell multi-tissue response function estimation, constrained spherical deconvolution, and tractogram generation with 107 output streamlines. The measure of structural connectivity was the number of fibers between pairs of regions divided by the sum of the volumes of the two regions. A group structural network was estimated by averaging all individuals’ structural networks. Functional volumes were spatially smoothed with a 5 mm isotropic Gaussian kernel and the first 10 volumes were discarded. fMRI time series were detrended and six head motion parameters, average cerebrospinal fluid, and white matter signal were regressed out. fMRI time series were band-pass filtered (0.01–0.15 Hz). fMRI data were parcellated according to the same atlas used for structural networks. Individual functional connectivity matrices were constructed by estimating the Pearson correlation between the fMRI time series of each pair of brain regions. A group-average functional connectivity matrix was estimated as the average connectivity between pairwise regions across individuals.

### Laplacian eigenmodes

To generate a dissociation of distinct diffusion processes, we performed the eigendecomposition of the structural Laplacian. Specifically, we expressed the structural connectome as an undirected, weighted adjacency matrix **A**. Then, the structural Laplacian can be defined as

1 $${{{{{\bf{L}}}}}}={{{{{\bf{D}}}}}}-{{{{{\bf{A}}}}}}$$

where **D** represents the diagonal weighted degree matrix. Following ref. ^61^, the structural Laplacian was normalized as ${{{{{{\bf{L}}}}}}}^{{\prime} }={{{{{\bf{L}}}}}}/{\lambda }_{\max }$ to preclude the influence of network sizes and densities, where ${\lambda }_{\max }$ indicated the largest eigenvalues of **L**. Through the eigendecomposition of the normalized structural Laplacian ${{{{{{\bf{L}}}}}}}^{{\prime} }{{{{{\bf{U}}}}}}={{{{{\bf{U}}}}}}{{{{{\boldsymbol{\Lambda }}}}}}$, we obtained a set of orthogonal eigenmodes ${{{{{{\bf{u}}}}}}}_{k}\epsilon {{{{{\bf{U}}}}}}{{{{{\boldsymbol{,}}}}}}\,k=1,\ldots,N,$ that correspond to distinct spatiotemporal patterns of signal propagation^32,55^. Their eigenvalues ${\lambda }_{k}\epsilon {{{{{\boldsymbol{\Lambda }}}}}}$ are closely related to persistent time and spatial complexity of spreading processes. Specifically, eigenmodes with near-zero eigenvalues sustain global and persistent diffusion patterns while eigenmodes with large eigenvalues capture geometrically complex spreading processes that delay quickly. Benefiting from their orthogonality, eigenmodes have been used as a parsimonious basis in the prediction of resting-state functional connectivity^34^.

### Regional structure-function prediction

The eigenmode approach is considered a powerful tool for structure-function prediction due to its appealing feature of representing the relationship simply and explicitly^36^. Functional connectivity matrix **F** can be interpreted as the aggregation of activating networked persistent modes captured by eigenmodes in appropriate proportion^31^, that is,

2 $$\hat{{{{{{\bf{F}}}}}}}={{{{{\bf{UC}}}}}}{{{{{{\bf{U}}}}}}}^{{{{{{\bf{T}}}}}}}=\mathop{\sum }\limits_{{{{{{\boldsymbol{k}}}}}}={{{{{\boldsymbol{1}}}}}}}^{{{{{{\boldsymbol{N}}}}}}}{c}_{k}{{{{{{\bf{u}}}}}}}_{k}{{{{{{\bf{u}}}}}}}_{k}^{T}$$

where **C** is a diagonal matrix with elements ${c}_{k}$ to be estimated.

The above formulation can also be derived from the network-diffusion model^32^

3 $$\frac{{{{{{\rm{d}}}}}}{{{{{\bf{x}}}}}}\left(t\right)}{{{{{{\rm{d}}}}}}t}=-\beta {{{{{\bf{Lx}}}}}}\left(t\right)$$

where **x**(*t*) denotes the time evolution of neural activity and parameter $\beta$ corresponds to the decay rate. It has the analytical solution ${{{{{\bf{x}}}}}}\left(t\right)={e}^{-\beta {{{{{\bf{L}}}}}}{{{{{\rm{t}}}}}}}{{{{{{\bf{x}}}}}}}_{0}$, where ${{{{{{\bf{x}}}}}}}_{0}$ denotes the initial configuration of the diffusion process. Under the hypothesis that the configuration at a critical time ${t}_{{crit}}$ evolving from an initial configuration with only region *i* active is simply the set of functional connectivity between region *i* and all other regions (i.e., the *i*th column of the functional connectivity matrix)^33,36^, the whole-brain functional connectivity matrix can be estimated as

4 $$\hat{{{{{{\bf{F}}}}}}}{{{{{\boldsymbol{=}}}}}}\exp \left(-\beta {{{{{\bf{L}}}}}}{t}_{{crit}}\right)$$

By eigendecompositing the matrix **L** into ${{{{{\bf{L}}}}}}={{{{{\bf{U}}}}}}{{{{{\boldsymbol{\Lambda }}}}}}{{{{{{\bf{U}}}}}}}^{{{{{{\bf{T}}}}}}}$, the above equation can be rewritten as $\hat{{{{{{\bf{F}}}}}}}{{{{{\boldsymbol{=}}}}}}{{{{{\bf{U}}}}}}{e}^{-\beta {{{{{\boldsymbol{\Lambda }}}}}}{t}_{{crit}}}{{{{{{\bf{U}}}}}}}^{{{{{{\bf{T}}}}}}}$. When generalizing unknown parameters ${e}^{-\beta {{{{{\boldsymbol{\Lambda }}}}}}{t}_{{crit}}}$ as a diagonal matrix **C**, we obtained the same formulation

5 $$\hat{{{{{{\bf{F}}}}}}}{{{{{\boldsymbol{=}}}}}}{{{{{\bf{UC}}}}}}{{{{{{\bf{U}}}}}}}^{{{{{{\bf{T}}}}}}}{{{{{\boldsymbol{=}}}}}}\mathop{\sum }\limits_{k=1}^{N}{c}_{k}{{{{{{\bf{u}}}}}}}_{k}{{{{{{\bf{u}}}}}}}_{k}^{T}$$

For regional structure-function prediction, we introduced regional heterogeneity and expressed the functional connectivity profile of a given node *i* as a weighted combination of eigenmodes, that is,

6 $${\hat{{{{{{\bf{F}}}}}}}}_{i}{{{{{\boldsymbol{=}}}}}}{{{{{\bf{U}}}}}}{{{{{{\bf{b}}}}}}}^{i}{{{{{\boldsymbol{=}}}}}}\mathop{\sum }\limits_{k=1}^{N}{b}_{k}^{i}{{{{{{\bf{u}}}}}}}_{k}$$

where ${\hat{{{{{{\bf{F}}}}}}}}_{i}$ indicates the *i*th column of the $\hat{{{{{{\bf{F}}}}}}}$ and ${{{{{{\bf{b}}}}}}}^{i}={({b}_{1}^{i},\ldots,{b}_{N}^{i})}^{T}$ is a vector of parameters that needs to be estimated.

This formulation can also be derived from the network-diffusion, that is,

7 $${\hat{{{{{{\bf{F}}}}}}}}_{i}={{{{{{\bf{U}}}}}}{{{{{{{\bf{C}}}}}}}^{i}{{{{{\bf{U}}}}}}}^{{{{{{\bf{T}}}}}}}e}_{i}=\mathop{\sum }\limits_{k=1}^{N}{c}_{k}^{i}{u}_{{ik}}{{{{{{\bf{u}}}}}}}_{k}={{{{{{\bf{Ub}}}}}}}^{i}$$

where ${e}_{i}$ denotes the cardinal unit vector in the *i*th direction and$\,{{{{{{\bf{b}}}}}}}^{i}={({b}_{1}^{i},\ldots,{b}_{N}^{i})}^{T}$ is the parameter vector.

### Multilinear model

We constructed two multilinear regression models, using low-frequency and high-frequency eigenmodes as predictors, to predict the functional connectivity profile of each brain region. Since the eigenvalues encode the natural frequencies of the spatiotemporal patterns captured by corresponding eigenmodes^34,55^, we extracted the eigenmodes in increasing and descending order of eigenvalues to constitute the low-frequency and high-frequency components, respectively. Considering that there is no general method to determine the threshold ${K}_{L}$ and ${K}_{H}$, we chose a default value (${K}_{L}$ = 14, ${K}_{H}=434$), which falls within the range of typical thresholds used in previous literature^11,36,97^, and performed the sensitivity analyses to confirm the robustness of results to threshold selection (Supplementary Fig. S6).

For a given node *i*, the multilinear models were expressed as

8 $${\hat{{{{{{\bf{F}}}}}}}}_{i}^{L}={b}_{1}^{i,L}{{{{{{\bf{u}}}}}}}_{1}+{{{{{{{\bf{U}}}}}}}^{L}{{{{{\bf{b}}}}}}}^{i,L}$$

9 $${\hat{{{{{{\bf{F}}}}}}}}_{i}^{H}={b}_{1}^{i,H}{{{{{{\bf{u}}}}}}}_{1}+{{{{{{{\bf{U}}}}}}}^{H}{{{{{\bf{b}}}}}}}^{i,H}$$

where ${{{{{{\bf{U}}}}}}}^{L}=({{{{{{\bf{u}}}}}}}_{2},\ldots,{{{{{{\bf{u}}}}}}}_{{K}_{L}})$ and ${{{{{{\bf{U}}}}}}}^{H}=({{{{{{\bf{u}}}}}}}_{{K}_{H}},\ldots,{{{{{{\bf{u}}}}}}}_{N})$ are low-frequency and high-frequency predictor variables. The first eigenmode ${{{{{{\bf{u}}}}}}}_{1}$ was isolated to constitute constant terms as it represents a trivial homogeneous pattern (constant across the brain). The dependent variable is the ith column of functional connectivity matrix. Parameters ${b}_{1}^{i,L}$, ${b}_{1}^{i,H}$, ${{{{{{\bf{b}}}}}}}^{i,L}={({b}_{2}^{i},\ldots,{b}_{{K}_{L}}^{i})}^{T}$, and ${{{{{{\bf{b}}}}}}}^{i,H}={({b}_{{K}_{H}}^{i},\ldots,{b}_{N}^{i})}^{T}$ can be estimated using ordinary least squares method (OLS). Local structure-function correspondence is quantified as the goodness of fit, which is computed as the Pearson correlation coefficient *R* between predicted and empirical functional connectivity profiles of brain regions.

### Hypothesis tests and null models

Throughout the paper, we conducted three main hypothesis tests to examine the regional pattern of structure-function coupling estimated by low/high-frequency eigenmodes. First, to examine whether structure-function coupling is network-specific, we constructed a null model that randomly permutated nodes’ assignment to seven functional networks (10,000 repetitions). This model embodies a null hypothesis that the observed average strength *R* of structure-function coupling in each network does not depend on the partition. The *P*-value was calculated as the proportion of simulated test statistics that are more extreme than the observed test statistic, and then corrected for multiple comparisons. The second one was constructed to examine whether the spatial pattern of structure-function coupling is correlated with the macroscale functional gradient spanning from unimodal to transmodal cortex. We applied a spin test, a widely used spatial permutation test with spatial autocorrelation preserved^48^. The last one was to examine whether the spatial correspondence between structure-function coupling and the functional gradient depends on network topology. The null model was constructed by randomly swapping pairs of edges, which destroyed the topological organization of the empirical structural connectome while preserving the degree sequence.

### Model comparison

To determine whether high-frequency eigenmodes supplement information from low-frequency eigenmodes to yield improved predictions, we compared the performance of prediction models comprising only low-frequency eigenmodes and those comprising both low-frequency and high-frequency eigenmodes. Given the large number of high-frequency eigenmodes, we performed least absolute shrinkage and selection operator (LASSO) regression, which may eliminate unimportant variables by penalizing regression coefficients. First, we randomly split individual subjects into a training set consisting of 80% of subjects and a test set consisting of the remaining 20% (100 repetitions). To select regularization parameters, we employed nested parameter optimization which is strictly limited to the training set. That is, we applied another 80–20 split (20 repetitions) to the training set, where we trained models with different regularization parameter on 80% of the training set (using group-average SC and FC matrices) and assessed the performance on the remaining 20% of the training set (using group-average SC and FC matrices), with the model performance evaluated by the Pearson correlation coefficient between predicted and empirical functional connectivity profiles. We then selected the regularization parameter with the best performance to train a LASSO model on all training samples and constructed the prediction model based on the preserved features. We strictly limited the parameter selection and training procedure to the training set and then compared the performance of prediction model with and without high-frequency features on the test set. In the above procedure, we used the R package (v.4.2.2) glmnet to implement LASSO regression^98^. We also considered the potential of eigenmode switching (e.g., a structural eigenmode ranked 900th in the training set might be ranked 901st in the test set) and aligned the order of eigenmodes in training and test sets by minimizing their angles to corresponding eigenmodes of group-consensus SC matrix derived from averaging all individuals^34^.

As a control, we performed phase-randomization of empirical eigenmodes while preserving the original spatial frequency (10 × 100 repetitions), generating three null benchmarks that correspond to prediction models comprising phase-randomized low-frequency eigenmodes, comprising phase-randomized high-frequency eigenmodes, and comprising empirical low-frequency and phase-randomized high-frequency eigenmodes. The results were illustrated in Supplementary Fig. S10, which suggests that high-frequency eigenmodes brought significantly more value than noise. Finally, in addition to the main analysis of LASSO regression only for high-frequency eigenmodes (which preserved all low-frequency eigenmodes in the model comparison), we performed LASSO regression separately for low-frequency model, high-frequency model, and low-high combined model in supplementary analyses, which may result in the elimination of low-frequency features. As illustrated in Supplementary Fig. S11, the results were largely unchanged. We also replicated the analyses using different choices high-frequency thresholds (Supplementary Fig. S12). The results were largely unchanged.

### Reporting summary

Further information on research design is available in the Nature Portfolio Reporting Summary linked to this article.

### Supplementary information

Supplementary Information

Reporting Summary

### Source data

Source Data

## Data Availability

The Lausanne (LAU) dataset is publicly available at https://zenodo.org/record/2872624#.YTR9lI4zaUl. The Human Connectome project (HCP) dataset is publicly available at https://db.humanconnectome.org/with the acceptance of HCP Open Access Data Use Terms. The cortical segmentation according to the Desikan-Killiany atlas could be implemented in FreeSurfer (http://surfer.nmr.mgh.harvard.edu). Lausanne anatomical atlases, including different spatial resolutions, are available at https://github.com/connectomicslab/connectomemapper3. Source data are provided with this paper.

## Code availability

For HCP, the diffusion and functional data were processed with the MRtrix3 package [http://www.mrtrix.org/] and SPM8 [https://www.fil.ion.ucl.ac.uk/spm/]. Matlab (R2020a) and R (version 4.2.2) code for the main results of this paper is publicly available at https://github.com/yaqiany/scfccoupling-high and https://zenodo.org/record/8255376. The original code of the spin test is publicly available at https://github.com/spin-test/spin-test^99^.
