# Supplementary material for: Enhanced brain structure-function tethering in transmodal cortex revealed by high-frequency eigenmodes
Source: Nat Commun. 2023 Oct 24;14:6744. doi: 10.1038/s41467-023-42053-4 (PMC10598018; doi:10.1038/s41467-023-42053-4)
Supplement: Supplementary file 1 — Supplementary Information [file 41467_2023_42053_MOESM1_ESM.pdf]

Supplementary Information

Supplementary Note 1

Analysis of individual subjects

To improve the signal-to-noise ratio, we initially performed our analysis on group-average structural and functional networks. Here, we sought to understand the regional patterns of structure-function coupling estimated by low-frequency and high-frequency eigenmodes from the perspective of individual subjects.

For low-frequency eigenmodes, we conducted the fitting procedure for every subject, which returns a matrix of coupling R whose size is [69 subjects × 1,000 regions] (Fig. 6a). As in the previous section, we found considerable variability across regions (one-way ANOVA R; F(999)=11.9; $p<10^{-15}$), confirming the regionally heterogeneous roles of low-frequency eigenmodes in local structure-function prediction. To visualize the spatial distribution of these results, we averaged over subjects and plotted the mean structure-function coupling R for each region (Fig. 6b). We found that the magnitude of structure-function coupling varied systematically across the cortex, with primary unimodal cortices exhibiting the higher prediction accuracies than transmodal association cortex.

To assess whether the contributions of low-frequency eigenmodes were concentrated within specific functional systems, we aggregated these R values by seven functional networks and compared the network-specific mean R with the null distribution generated by a spatially-constrained permutation model (spin test; 10,000 permutations). We found that the visual network had significantly higher structure-function coupling relative to the null distribution while ventral attention, frontoparietal, and default mode networks exhibited lower R values than the level expected by chance (FDR corrected $P<10^{-4}$; Fig. 6c). Considering the inter-individual heterogeneity, we also provided the distributions of network-specific mean R over all subjects (Fig. S2a). Aggregating across subjects, we found that structure and function were gradually decoupled from the visual network (R=0.33±0.07) to the frontoparietal (R=0.24±0.05) and default mode networks (R=0.25±0.05). We further repeated the analysis of network-specific effects at a single subject level and calculated for each functional network the fraction of subjects for whom the network-specific mean R was statistically significantly different from the null distribution generated by spatial permutation (FDR corrected $P<10^{-4}$). As expected, we found that the visual network in 93% of subjects exhibited statistically significantly higher R values compared to the null distribution while the frontoparietal network in 43% of subjects and the default mode network in 71% of subjects exhibited statistically significantly lower R values than expected by chance.

We then correlated the regional coupling R with the unimodal-transmodal functional gradient for every subject, comparing the empirical correlation coefficient against those obtained using a spatially-constrained permutation model (1,000 permutations) and against those generated by randomly rewiring network edges with degree sequence preserved (1,000 permutations). Fig. S3 shows the spatial distributions of regional coupling R (panel a) for three individual subjects, as well as scatter plots showing the correlation between the functional gradient and coupling R (panel b) and the two null distributions for this correlation (panel c and d). In general, we found that the correlation between the regional R and functional gradient was overall negative although these correlation coefficients were considerably variable across subjects (Pearson ρ=-0.297±0.168; Fig. S2b), implying that structure and

function are gradually divergent along the unimodal-transmodal hierarchy. Using the above two kinds of subject-specific null models, we found that this anticorrelation was statistically significant in 88% of subjects relative to the first null distribution and in 71% of subjects relative to the second null distribution ($P<0.05$).

For high-frequency eigenmodes, the structure-function coupling estimated at the individual level yielded a matrix containing R of every region and subject, which was reported in Fig. 6d. Consistent with the previous section, we observed regionally heterogeneous contributions of high-frequency eigenmodes to structure-function prediction (one-way ANOVA R; F(999)=15.7; $P<10^{-15}$). Averaging regional R across subjects, we observed relatively weak structure-function coupling in unimodal primary and particularly in visual regions, and relatively strong coupling in transmodal association regions (Fig. 6d).

To examine whether the contributions of high-frequency eigenmodes are system-specific, we aggregated these R values by seven functional networks, comparing the network-specific mean R with those obtained by the spatially-constrained permutation model (spin test; 10,000 permutations). We found that regions with lower R values were prominent in the visual and dorsal attention networks whereas regions with higher R values were affiliated with the limbic, frontoparietal, and default mode networks (FDR corrected $P<10^{-4}$; Fig. 6f). We also took into account inter-individual heterogeneity and plotted the distribution of network-specific mean R over all subjects (Fig. S2c). In general, the strength of structure-function coupling increased from the visual network (R=0.58±0.03) to the default mode networks (R=0.62±0.02). Compared to the null distributions generated using the spatially-constrained permutation model (spin test; 10,000 permutations), we found that 84% of subjects exhibited statistically significantly lower R values in the visual network (FDR corrected $P<10^{-4}$). In contrast, the default mode network in 41% of subjects, the frontoparietal network in 23% of subjects, and the limbic network in 32% of subjects displayed statistically significantly higher R values than expected by chance (FDR corrected $P<10^{-4}$). These results demonstrate that the magnitude of coupling R estimated by high-frequency eigenmodes was circumscribed by functional systems, with visual cortices showing relatively lower structure-function coupling than association cortices.

Furthermore, we estimated the correlation between the regional structure-function coupling R and the unimodal-transmodal functional gradient for every subject. The examples of individuals' spatial distributions of R were provided in Fig. S4 (panel a), with their corresponding correlations to the macroscale functional gradient illustrated in Fig. S4 (panel b). In general, we found that the regional R and functional gradient were overall positively correlated across subjects (Pearson ρ=0.254±0.157; Fig. S2d), implying that local structure-function relationships estimated by high-frequency eigenmodes are increasingly convergent along the unimodal-transmodal hierarchy. For every subject, we also compared the empirical correlation coefficient against those generated by two kinds of null models (Fig. S4, panel c and d). In the first one, we performed spatial permutation with spatial autocorrelation preserved (spin test; 1,000 permutations); in the second one, we rewired structural connections but preserved the original degree sequence (1,000 permutations). We found that 87% of subjects exhibited statistically significantly higher correlation coefficients than the first null distribution and that 65% of subjects than the second null distribution ($P<0.05$).

Finally, we estimated the standard deviation of coupling R estimated by low-frequency and high-frequency eigenmodes for every region across all subjects. Interestingly, we found that regions with great inter-individual variations were overall concentrated in the visual and somatosensory cortex

whereas structure-function coupling in prefrontal, lateral temporal, and inferior parietal cortex was relatively consistent across subjects (Fig. S5a and c). Note that, for low-frequency eigenmodes, this spatial pattern is very similar to the spatial distribution of regional coupling R (Pearson ρ=0.79), and when transforming the standard deviation to the coefficient of variation, this trend did not persist (Pearson ρ=0.05; P=0.09; Fig. S5b), suggesting that the regional heterogeneity in inter-individual variability observed in the case of low-frequency eigenmodes may be attributable to a floor effect. In contrast, for high-frequency eigenmodes, the negative association between inter-individual variation in structure-function coupling and the unimodal-transmodal functional gradient was still statistically significant (Pearson ρ=-0.53; $P<10^{-5}$; Fig. S5d).

Collectively, these results suggest that the contribution of low-frequency eigenmodes is not uniform across the brain but concentrated on the primary unimodal regions, resulting in structure-function decoupling along the unimodal-transmodal gradient. Conversely, high-frequency eigenmodes preferentially contributed to the interpretation of functional profiles of transmodal association regions, inducing gradually convergent structure-function relationships from unimodal to transmodal regions. Both of these results are consistent with those obtained from group-average data and motivate further investigation into regional patterns of structure-function tethering under different diffusion processes.

Supplementary Note 2

Sensitivity analyses

we performed several sensitivity analyses to confirm the robustness of the regional structure-function coupling results to choices of low-frequency and high-frequency thresholds, spatial resolutions, data acquisition, and network reconstruction. First, we calculated regional structure-function coupling using low-frequency and high-frequency eigenmodes under the choice of K_L ranging from K_L=10 to K_L=20 and K_H ranging from K_H=384 to K_H=484, respectively. We found that the regional patterns of structure-function coupling were highly robust to the choice of thresholds (the mean spatial correlation was 0.99 (S.D. 0.02) for low frequencies and 0.99 (S.D. 0.00) for high frequencies; Supplementary Fig. S6). We also observed good agreement with the main regional coupling patterns when using structural and functional networks derived: (1) at another four resolutions (68, 114, 219, 448 nodes; Supplementary Fig. S7) and (2) using an independently collected dataset (HCP; Supplementary Fig. S8). We further repeated our analyses using functional networks derived from the partial correlation and found comparable results with the main text (Supplementary Fig. S9).

Supplementary Note 3

Comparison of prediction models with and without high-frequency eigenmodes

Null models

As a control, we performed phase-randomization of empirical eigenmodes while preserving the original spatial frequency to build null benchmarks (10×100 repetitions) [1-3]. We generated three null distributions, which corresponded to prediction models comprising phase-randomized low-frequency eigenmodes, comprising phase-randomized high-frequency eigenmodes, and comprising empirical low-frequency and phase-randomized high-frequency eigenmodes. We found that both prediction models

containing only low-frequency eigenmodes and containing only high-frequency eigenmodes significantly outperformed the corresponding null models ($P<10^{-3}$; Fig. S10a-c), confirming the contribution of low-frequency and high-frequency eigenmodes to structure-function prediction. We further combined empirical low-frequency eigenmodes with phase-randomized high-frequency eigenmodes and compared this null benchmark with the empirical combined model to determine whether high-frequency eigenmodes supplement information from low-frequency eigenmodes to yield improved predictions. By calculating the change in prediction accuracy (ΔR), we found that the null benchmark underperformed the low-high combined model, and even the baseline low-frequency model (in both cases $P<10^{-3}$; Fig. S10d). Thus, while low-frequency eigenmodes could well predict functional connectivity on their own, the introduction of noise would significantly hurt the performance of pre-existing model with good features. The small but statistically significant improvement from combining low-frequency and high-frequency eigenmodes strongly suggests this is not the case with high-frequency eigenmodes. Instead, the addition of high-frequency eigenmodes enhanced the explanation of functional interaction patterns, suggesting that high-frequency eigenmodes brought significantly more value than noise.

Lasso regression for all three prediction models

In the main text, we performed LASSO regression only for high-frequency eigenmodes and preserved all low-frequency eigenmodes for model comparison. Here, we performed LASSO regression for low-frequency model, high-frequency model, and low-high combined model, separately. The results were illustrated in Fig. S11. We found that low-high combined outperformed low-frequency model and that the top 10% of brain regions with the highest percentage increases were mostly located in transmodal association areas.

Robustness to high-frequency threshold

In the main text, we report results using the high-frequency threshold K_H=434. Here, we extended the choice of K_H to a wide range (from the highest 200 modes to the highest 600 modes) and repeated the main analyses of the manuscript. We found that the observed prediction improvements and preference for transmodal regions were stable across different threshold choices (Fig. S12).

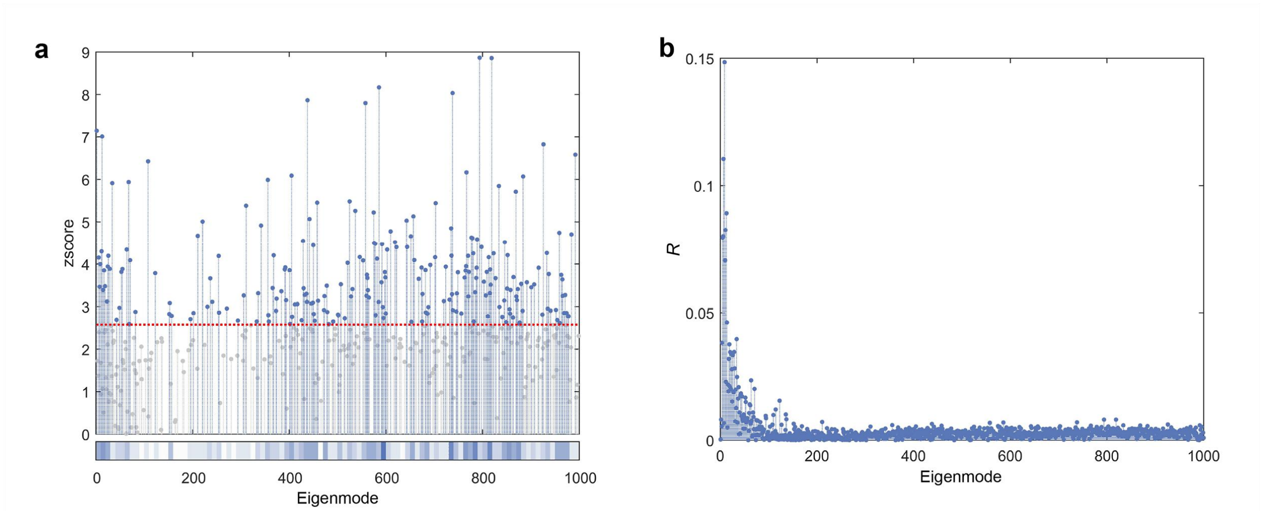

Fig. S1.

Prediction performance of single eigenmodes. (**a**) We applied each eigenmode to whole-brain structure-function prediction and expressed the prediction accuracy *R* into a z score relative to the null distribution generated by the corresponding phase-randomized eigenmodes (10,000 repetitions). Statistically significant eigenmodes were shown in color (one-sided $P<0.005$). (**b**) The whole-brain prediction accuracy *R* estimated by each individual eigenmode. Source data are provided as a Source Data file.

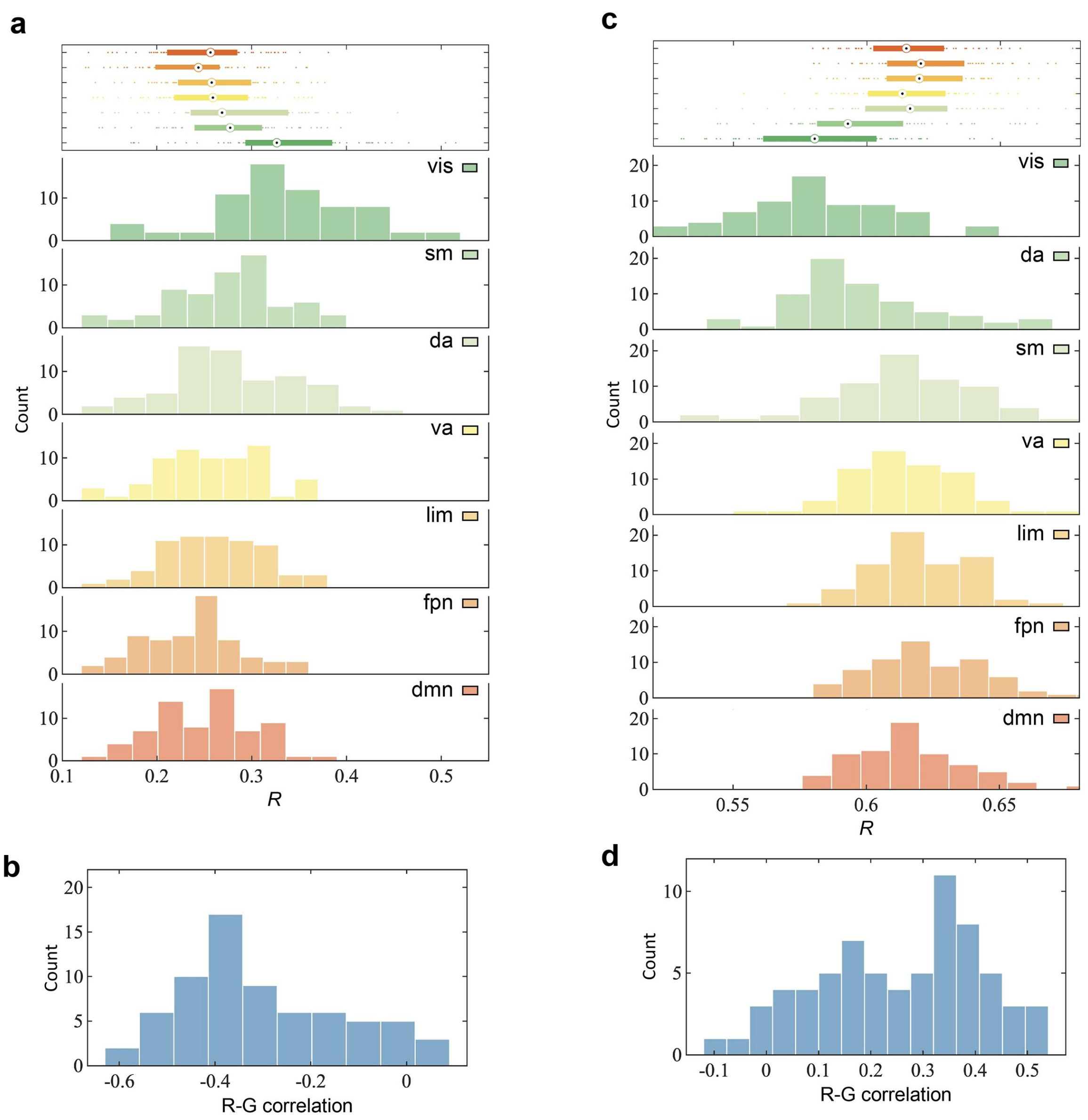

Fig. S2.

Individual structure-function coupling. (**a**) The distribution of network-specific mean *R* estimated by low-frequency eigenmodes over all subjects (n=69 subjects). Seven resting-state networks (RSNs): visual (vis), somatomotor (sm), dorsal attention (da), frontoparietal (fpn), ventral attention(va), limbic (lim), and default mode (dmn) networks. The boxplot shows the medians (circles), interquartile ranges (boxes), and min to max range (whiskers). (**b**) The histogram of correlation coefficients between regional coupling *R* estimated by low-frequency eigenmodes and functional gradient across all subjects. (**c**) The distribution of network-specific mean *R* estimated by high-frequency eigenmodes over all subjects (n=69 subjects). The boxplot shows the medians (circles), interquartile ranges (boxes), and min to max range (whiskers). (**d**) The histogram of correlation coefficients between regional coupling *R* estimated by high-frequency eigenmodes and functional gradient across all subjects. Source data are provided as a Source Data file.

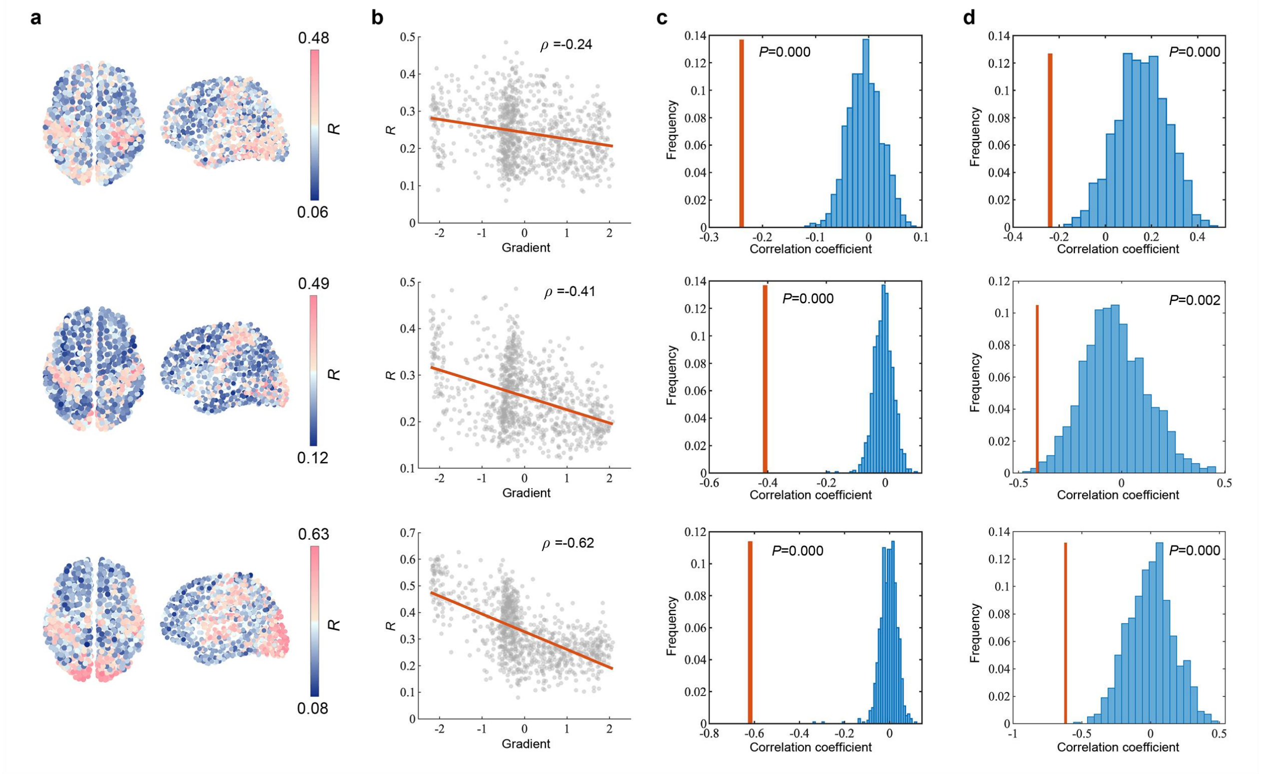

Fig. S3.

Regional structure-function coupling estimated by low-frequency eigenmodes for three individual subjects. (**a**) The spatial distribution. (**b**) The scatter plot showing the correlation between the functional gradient and regional coupling *R*. (**c**) Comparison of the empirical correlation coefficient against the null distribution generated by spatially-constrained permutation (1,000 repetitions). (**d**) Comparison of the empirical correlation coefficient against the null distribution generated by randomly rewiring network edges (1,000 repetitions). In (**c**) and (**d**), we provided the empirical P-values (one-sided, unadjusted), calculated as the proportion of correlation coefficients generated by the null model that were more extreme than the empirical correlation coefficients. Source data are provided as a Source Data file.

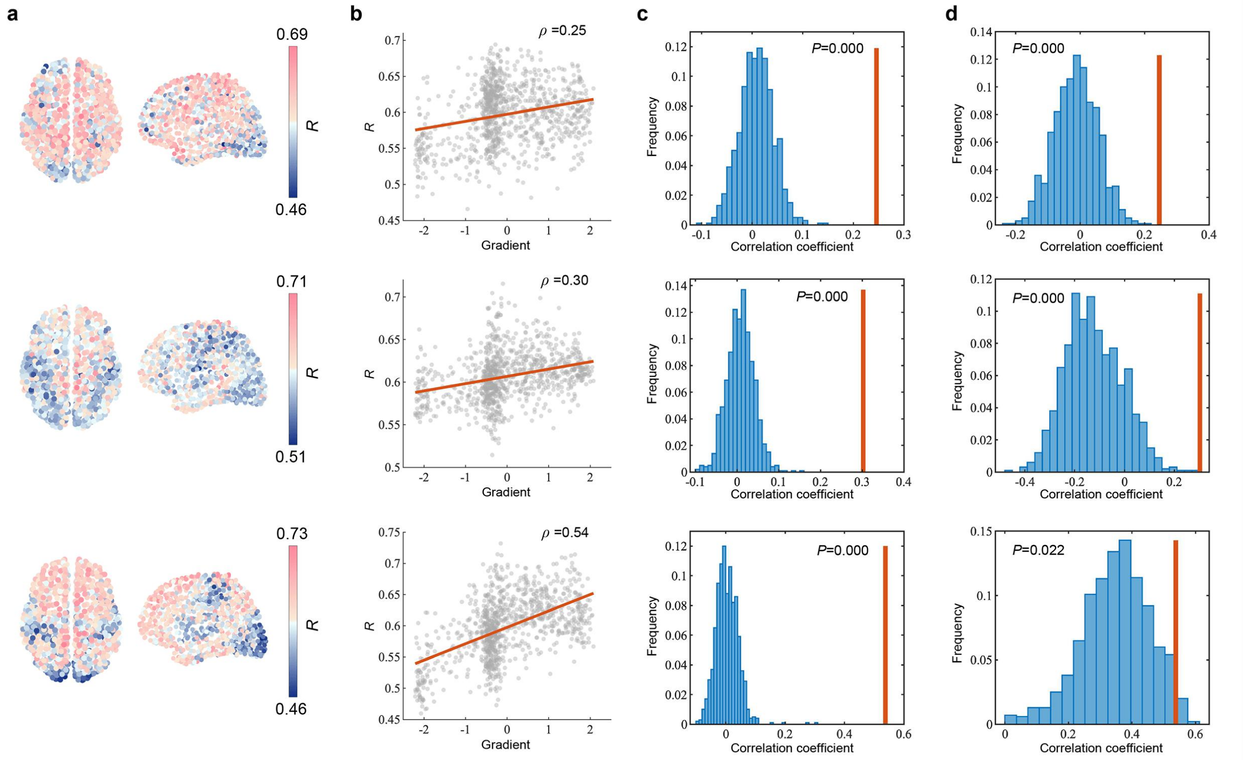

Fig. S4.

Regional structure-function coupling estimated by high-frequency eigenmodes for three individual subjects. (**a**) The spatial distribution. (**b**) The scatter plot showing the correlation between the functional gradient and regional coupling *R*. (**c**) Comparison of the empirical correlation coefficient against the null distribution generated by spatially-constrained permutation (1,000 repetitions). (**d**) Comparison of the empirical correlation coefficient against the null distribution generated by randomly rewiring network edges (1,000 repetitions). In (**c**) and (**d**), we provided the empirical P-values (one-sided, unadjusted). Source data are provided as a Source Data file.

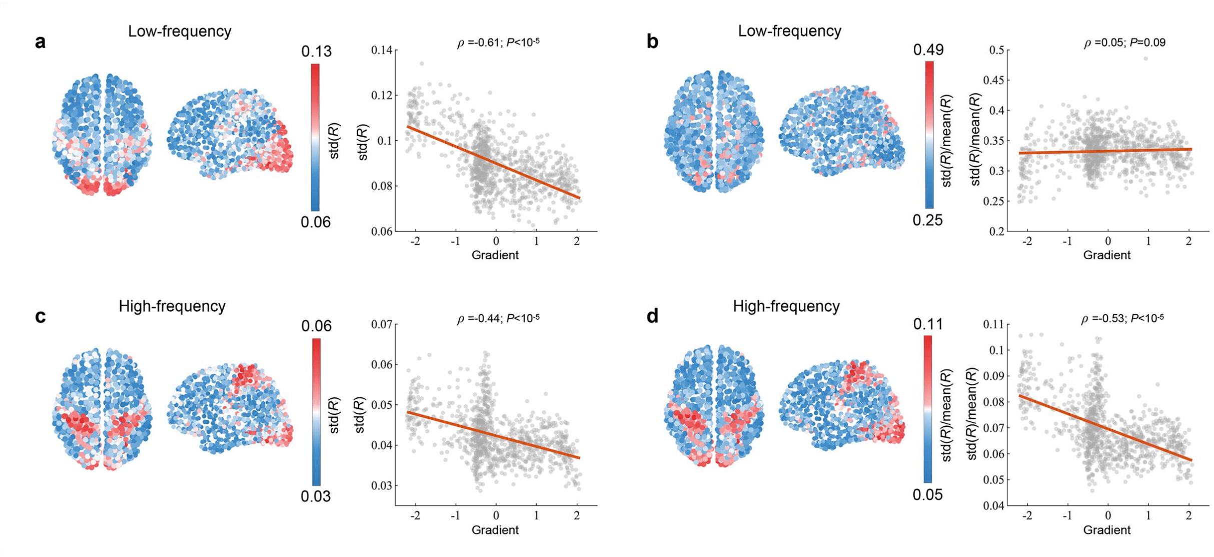

Fig. S5.

Inter-individual heterogeneity. (**a**) The standard deviation of regional coupling (n=1,000 regions) estimated by low-frequency eigenmodes across all subjects (left), which is negatively correlated with the unimodal-transmodal gradient (right). (**b**) When transforming the standard deviation to the coefficient of variation, this negative correlation did not persist. (**c**) The standard deviation of regional coupling (n=1,000 regions) estimated by high-frequency eigenmodes across all subjects (left), which is negatively correlated with the unimodal-transmodal gradient (right). (**d**) When transforming the standard deviation to the coefficient of variation, this negative correlation was still statistically significant. In (**a**)-(**d**), two-sided t-test p-values were calculated. Source data are provided as a Source Data file.

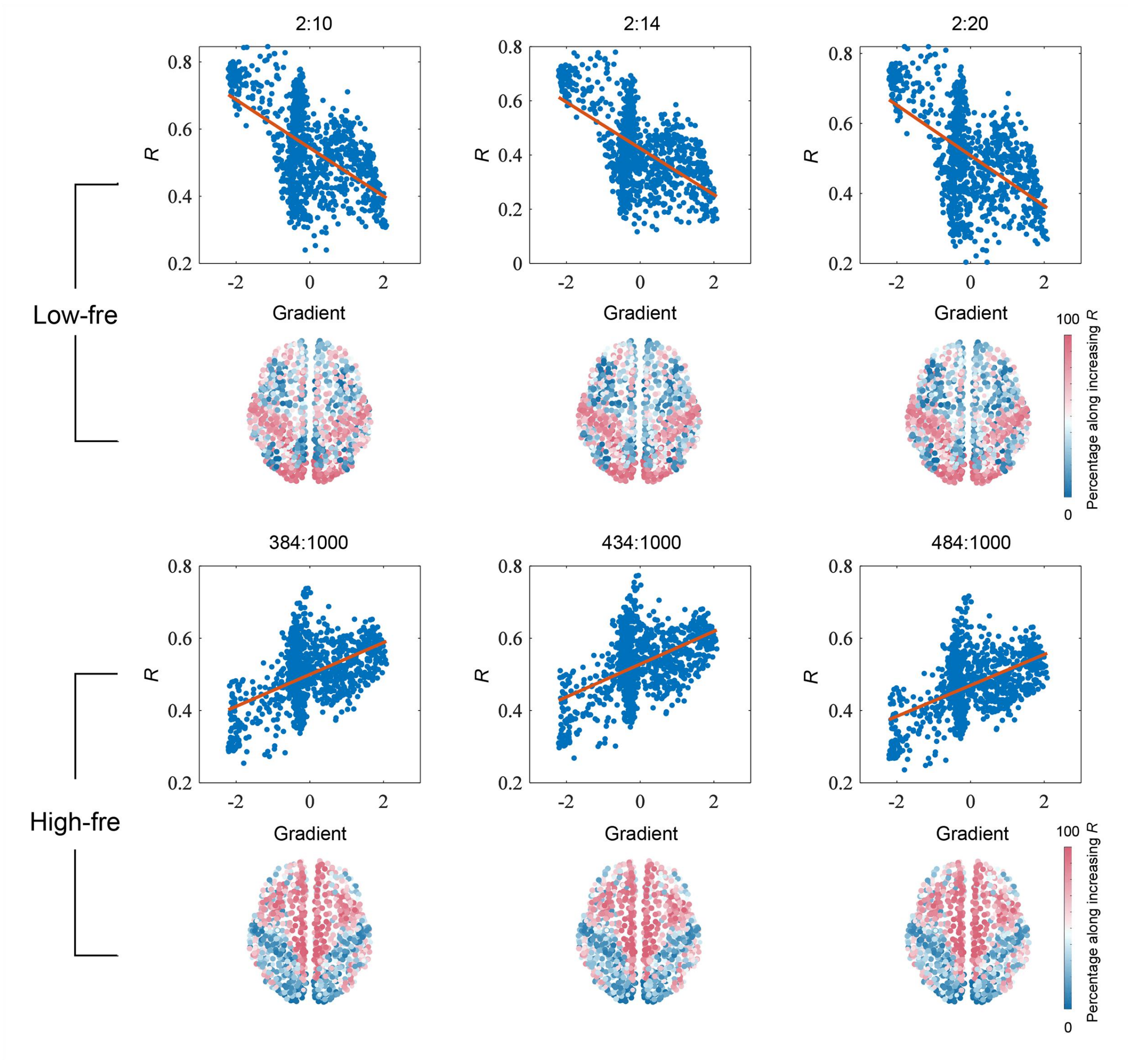

Fig. S6.

Robustness to frequency thresholds. The relationship between coupling strength *R* and the functional gradient is stable under different definitions of low-frequency and high-frequency eigenmodes. Source data are provided as a Source Data file.

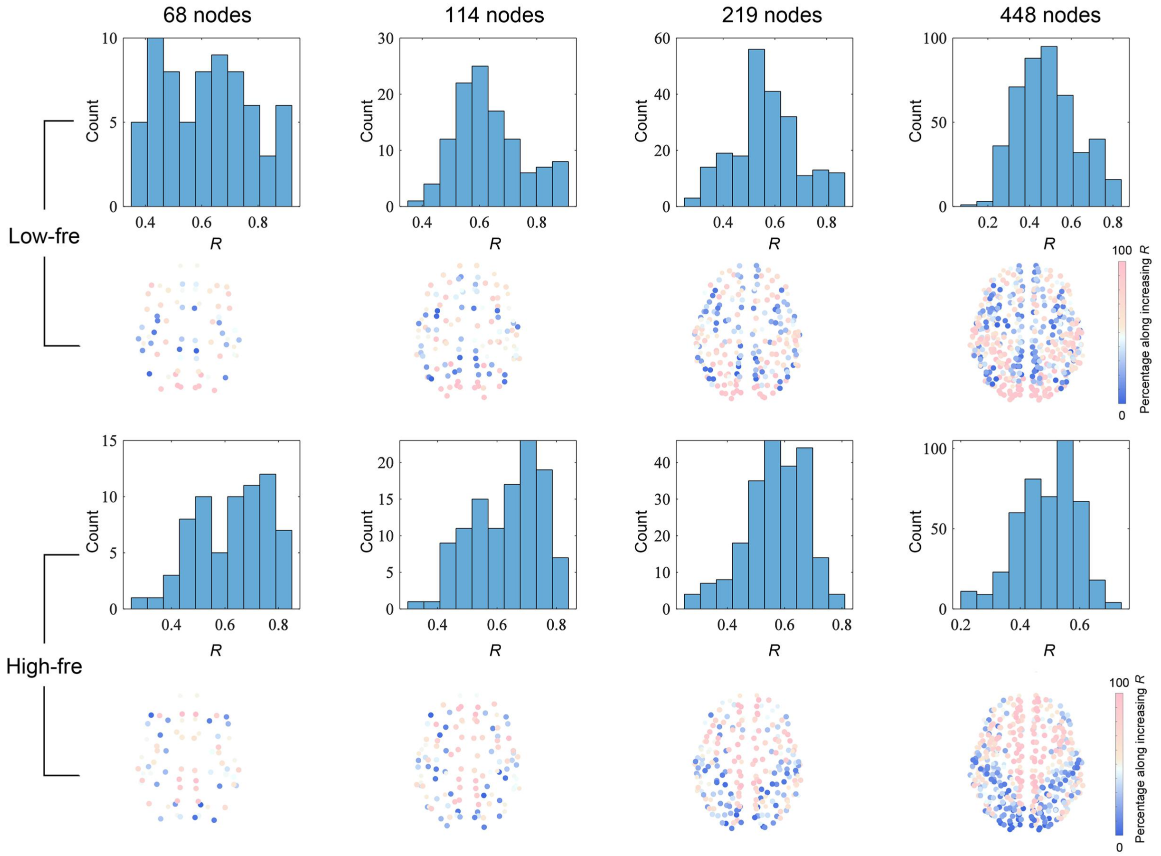

Fig. S7.

Consistency across spatial resolutions. The node-wise structure-function predictions based on low-frequency and high-frequency eigenmodes are respectively repeated in another four spatial resolutions (68, 114, 219, 448 nodes). The spatial patterns of structure-function *R* are visually similar. Source data are provided as a Source Data file.

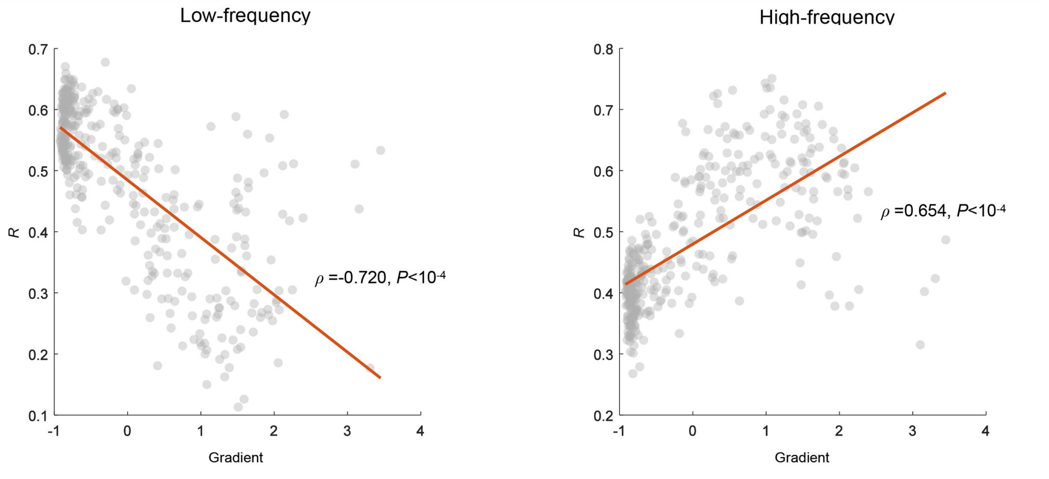

Fig. S8.
Verification on an independent dataset. The main results (structure-function divergence and convergence along the unimodal-transmodal gradient) are replicated in an independently collected dataset (Human Connectome Project HCP). Two-sided t-test p-values were calculated. Source data are provided as a Source Data file.

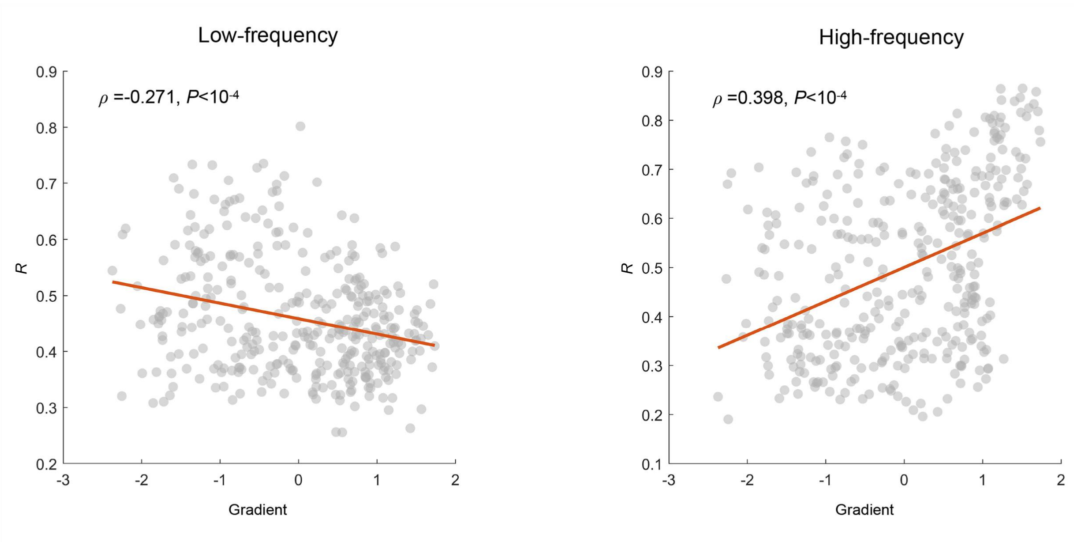

Fig. S9.

Robustness to network reconstruction. The main results (structure-function divergence and convergence along the unimodal-transmodal gradient) are preserved when using the functional connectivity matrix derived from partial correlation. Two-sided t-test p-values were calculated. Source data are provided as a Source Data file.

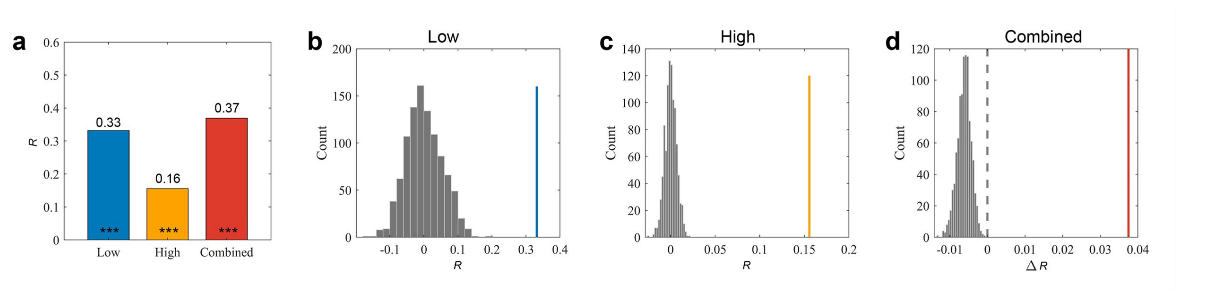

Fig. S10.

Comparison to null models. (**a**) The prediction performances of low-frequency eigenmodes (blue), high-frequency eigenmodes (orange) and low-high combined model (red) across the brain, which significantly outperform the null distributions obtained from phase-randomized low-frequency eigenmodes, phase-randomized high-frequency eigenmodes, and the model combining low and phase-randomized high eigenmodes (*** the empirical $P<10^{-3}$). (**b**) Blue: the performance of prediction model comprising empirical low-frequency eigenmodes. Grey: the null distribution generated by prediction model comprising phase-randomized low-frequency eigenmodes. (**c**) Orange: the performance of prediction model comprising empirical high-frequency eigenmodes. Grey: the null distribution generated by prediction model comprising phase-randomized high-frequency eigenmodes. (**d**) Red: the increment in prediction accuracy (ΔR) with the addition of empirical high-frequency eigenmodes. Grey: the null distribution of changes in prediction accuracy generated by combining empirical low-frequency eigenmodes with phase-randomized high-frequency eigenmodes. Source data are provided as a Source Data file.

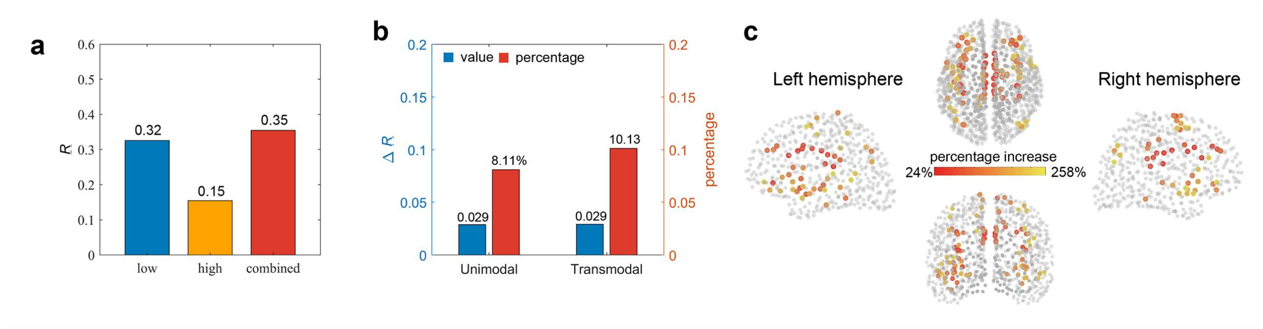

Fig. S11.

LASSO regression for all three prediction models. We performed Lasso regression separately for low-frequency model, high-frequency model, and low-high combined model, which may result in the elimination of low-frequency features. (**a**) The prediction performances of low-frequency eigenmodes (blue), high-frequency eigenmodes (orange) and low-high combined model (red). (**b**) The increments (blue) and increase percentages (red) in prediction accuracy with the addition of high-frequency eigenmodes for unimodal and transmodal regions. (**c**) The spatial distribution of the top 10% of nodes with the highest percentage increases in prediction accuracy. Source data are provided as a Source Data file.

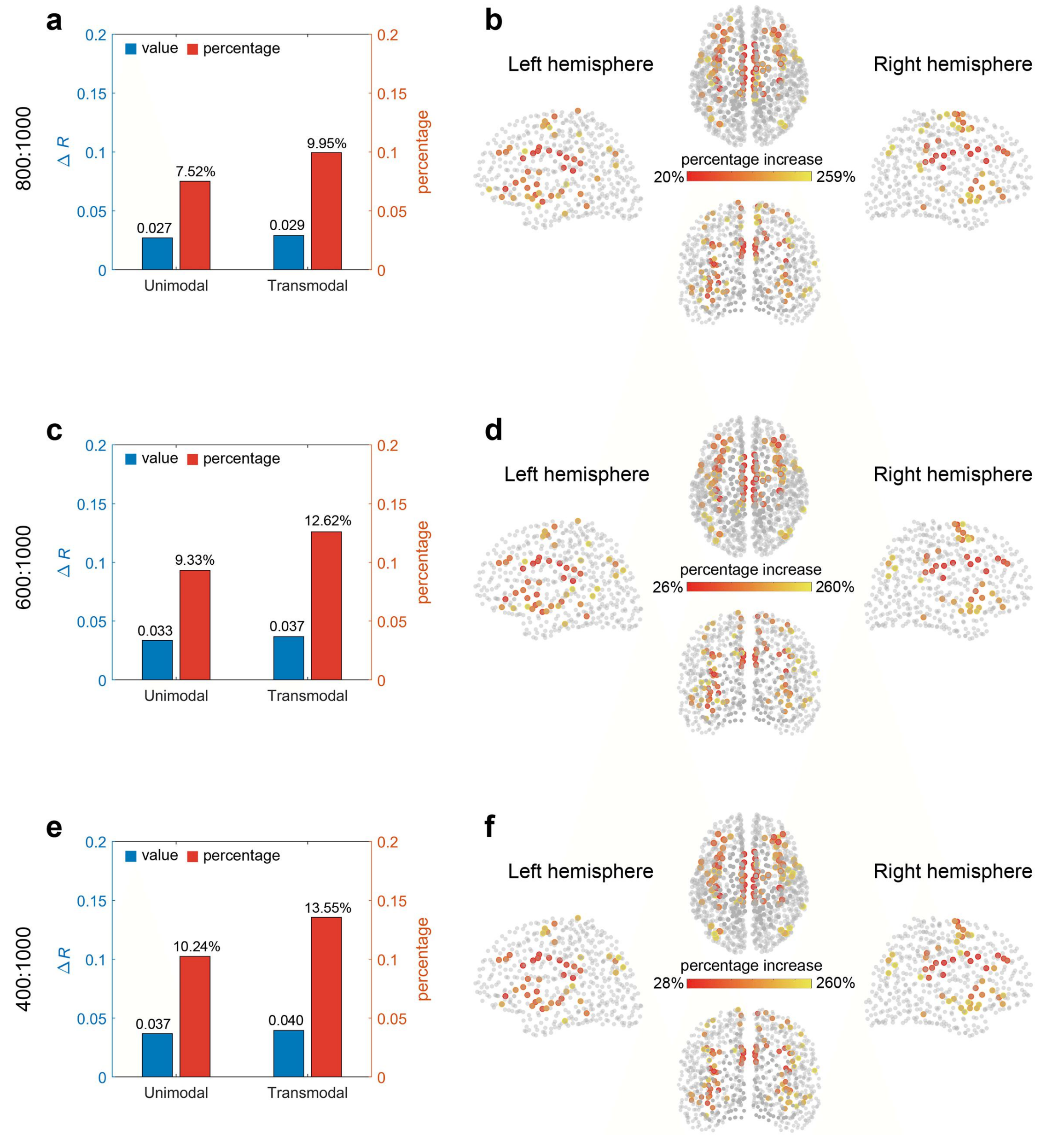

Fig. S12.

Replication of main results under different high-frequency thresholds. Left panel: the increment (blue) and increase percentage (red) in prediction accuracy with the addition of high-frequency eigenmodes for unimodal and transmodal region. Right panel: The spatial distribution of the top 10% of nodes with the highest percentage increases in prediction accuracy. (**a-b**) Results of high-frequency eigenmodes defined as 800:1000 modes. (**c-d**) Results of high-frequency eigenmodes defined as 600:1000 modes. (**e-f**) Results of high-frequency eigenmodes defined as 400:1000 modes. Source data are provided as a Source Data file.

Supplementary References

1. Tewarie, P., Bright, M. G., Hillebrand, A., Robson, S. E., Gascoyne, L. E., Morris, P. G., ... & Brookes, M. J. (2016). Predicting haemodynamic networks using electrophysiology: The role of non-linear and cross-frequency interactions. Neuroimage, 130, 273-292.
2. Hunt, B. A., Tewarie, P. K., Mougin, O. E., Geades, N., Jones, D. K., Singh, K. D., ... & Brookes, M. J. (2016). Relationships between cortical myeloarchitecture and electrophysiological networks. Proceedings of the National Academy of Sciences, 113(47), 13510-13515.
3. Tewarie, P., Abeysuriya, R., Byrne, Á., O'Neill, G. C., Sotiropoulos, S. N., Brookes, M. J., & Coombes, S. (2019). How do spatially distinct frequency specific MEG networks emerge from one underlying structural connectome? The role of the structural eigenmodes. NeuroImage, 186, 211-220.
