# Supplementary material for: Enhanced brain structure-function tethering in transmodal cortex revealed by high-frequency eigenmodes
Source: Nat Commun. 2023 Oct 24;14:6744. doi: 10.1038/s41467-023-42053-4 (PMC10598018; doi:10.1038/s41467-023-42053-4)
Supplement: Supplementary file 2 — Reporting Summary [file 41467_2023_42053_MOESM2_ESM.pdf]

nature portfolio

Corresponding author(s): Xin Wang, Shaoting Tang

Last updated by author(s): Aug 17, 2023

Reporting Summary

Nature Portfolio wishes to improve the reproducibility of the work that we publish. This form provides structure for consistency and transparency in reporting. For further information on Nature Portfolio policies, see our Editorial Policies and the Editorial Policy Checklist.

Statistics

For all statistical analyses, confirm that the following items are present in the figure legend, table legend, main text, or Methods section.

n/a	Confirmed	
☐	☒	The exact sample size (*n*) for each experimental group/condition, given as a discrete number and unit of measurement
☐	☒	A statement on whether measurements were taken from distinct samples or whether the same sample was measured repeatedly
☐	☒	The statistical test(s) used AND whether they are one- or two-sided<br>*Only common tests should be described solely by name; describe more complex techniques in the Methods section.*
☒	☐	A description of all covariates tested
☐	☒	A description of any assumptions or corrections, such as tests of normality and adjustment for multiple comparisons
☐	☒	A full description of the statistical parameters including central tendency (e.g. means) or other basic estimates (e.g. regression coefficient) AND variation (e.g. standard deviation) or associated estimates of uncertainty (e.g. confidence intervals)
☐	☒	For null hypothesis testing, the test statistic (e.g. *F*, *t*, *r*) with confidence intervals, effect sizes, degrees of freedom and *P* value noted<br>*Give P values as exact values whenever suitable.*
☒	☐	For Bayesian analysis, information on the choice of priors and Markov chain Monte Carlo settings
☒	☐	For hierarchical and complex designs, identification of the appropriate level for tests and full reporting of outcomes
☐	☒	Estimates of effect sizes (e.g. Cohen's *d*, Pearson's *r*), indicating how they were calculated

Our web collection on statistics for biologists contains articles on many of the points above.

Software and code

Policy information about availability of computer code

Data collection	No software was used for data collection.
Data analysis	For HCP, the diffusion and functional data were processed with the MRtrix3 package [http://www.mrtrix.org/] and SPM8 [https://www.fil.ion.ucl.ac.uk/spm/]. Matlab (R2020a) and R (version 4.2.2) code for the main results of this paper is publicly available at https://github.com/yaqiany/scfccoupling-high and https://zenodo.org/record/8255376. The original code of the spin test is publicly available at https://github.com/spin-test/spin-test.

For manuscripts utilizing custom algorithms or software that are central to the research but not yet described in published literature, software must be made available to editors and reviewers. We strongly encourage code deposition in a community repository (e.g. GitHub). See the Nature Portfolio guidelines for submitting code & software for further information.

Data

Policy information about availability of data

All manuscripts must include a data availability statement. This statement should provide the following information, where applicable:

- Accession codes, unique identifiers, or web links for publicly available datasets
- A description of any restrictions on data availability
- For clinical datasets or third party data, please ensure that the statement adheres to our policy

The Lausanne (LAU) dataset is publicly available at https://zenodo.org/record/2872624#.YTR9lI4zaUl. The Human Connectome project (HCP) dataset is publicly

available at https://db.humanconnectome.org/with the acceptance of HCP Open Access Data Use Terms. The cortical segmentation according to the Desikan-Killiany atlas could be implemented in FreeSurfer (http://surfer.nmr.mgh.harvard.edu). Lausanne anatomical atlases, including different spatial resolutions, are available at https://github.com/connectomicslab/connectomemapper3.

Human research participants

Policy information about studies involving human research participants and Sex and Gender in Research.

Reporting on sex and gender	LAU: M/F 43/27.<br>HCP: M/F 22/34.<br>Since the authors of the present study were not involved in the recruitment of subjects, we have no idea whether sex and/or gender of participants was determined based on self-report or assigned (and methodology used). No sex- and gender-based analyses have been performed because the sample size is low and we did not obtain information on individuals' sex or gender.
Population characteristics	For LAU, the dataset was collected from a cohort of 70 participants (28.8±9.1 years old). For HCP, the dataset consisted of 56 participants (26-35 years old).
Recruitment	The authors of the present study were not involved in the recruitment. Therefore, the authors are not knowledgeable about possible self-section biases or other biases associated with the data collection of the LAU and HCP cohorts. Given the consistency of the results across the two datasets, we would estimate that potential biases related to recruitment would not likely impact the main results of the study.
Ethics oversight	LAU was approved by the Ethics Committee of Clinical Research of the Faculty of Biology and Medicine, University of Lausanne. HCP was approved by the Washington University institutional review board.

Note that full information on the approval of the study protocol must also be provided in the manuscript.

Field-specific reporting

Please select the one below that is the best fit for your research. If you are not sure, read the appropriate sections before making your selection.

☒ Life sciences ☐ Behavioural & social sciences ☐ Ecological, evolutionary & environmental sciences

For a reference copy of the document with all sections, see nature.com/documents/nr-reporting-summary-flat.pdf

Life sciences study design

All studies must disclose on these points even when the disclosure is negative.

Sample size	Two independent datasets were used in this study. The main dataset was collected by Department of Radiology, University Hospital Center and University of Lausanne (LAU). The sample size of this dataset was determined by the LAU release, which contained 70 participants. The other is from the the Human Connectome Project (HCP; n=56), which favors the reliability of our results. No statistical methods were used to predetermine sample sizes. The replicability of our main results across the two independently collected datasets as well as across individual subjects demonstrate the stability of our findings.
Data exclusions	No data were excluded from the analysis.
Replication	The results were replicable across 5 spatial resolutions (68, 114, 219, 448, 1000 nodes) and two independently collected datasets (LAU, HCP).
Randomization	Subjects were not partitioned into groups. Data from each dataset (LAU, HCP) were analyzed separately so as to not mix data across acquisition machines and acquisition parameters.
Blinding	No blinding was applied to this study. As the study only used resting-state data and there was no experimental manipulation, blinding was not relevant to the analyses reported in this study .

Reporting for specific materials, systems and methods

We require information from authors about some types of materials, experimental systems and methods used in many studies. Here, indicate whether each material, system or method listed is relevant to your study. If you are not sure if a list item applies to your research, read the appropriate section before selecting a response.

Materials & experimental systems

n/a	Involved in the study	
☒	☐	Antibodies
☒	☐	Eukaryotic cell lines
☒	☐	Palaeontology and archaeology
☒	☐	Animals and other organisms
☒	☐	Clinical data
☒	☐	Dual use research of concern

Methods

n/a	Involved in the study	
☒	☐	ChIP-seq
☒	☐	Flow cytometry
☐	☒	MRI-based neuroimaging

Magnetic resonance imaging

Experimental design

Design type: Resting state

Design specifications: For LAU, 280 functional images, 128 diffusion-weighted volumes and a single b0 volume acquired per subject.
For HCP, 1200 frames acquired per session, four sessions of 14.4 minutes included.

Behavioral performance measures: No behavioral performance measures

Acquisition

Imaging type(s): functional, structural, diffusion

Field strength: 3T

Sequence & imaging parameters: For LAU, Diffusion spectrum images (DSI) were acquired on a 3-Tesla MRI scanner (Trio, Siemens Medical, Germany) using a 32-channel head-coil. The protocol was comprised of (1) a magnetization-prepared rapid acquisition gradient echo (MPRAGE) sequence sensitive to white/gray matter contrast (1-mm in-plane resolution, 1.2-mm slice thickness), (2) a DSI sequence (128 diffusion-weighted volumes and a single b0 volume, maximum b-value 8,000 s/mm2, 2.2×2.2×3.0 mm voxel size), and (3) a gradient echo EPI sequence sensitive to blood oxygen level-dependent (BOLD) contrast (3.3-mm in-plane resolution and slice thickness with a 0.3-mm gap, TR 1,920 ms, resulting in 280 images per participant).
For HCP, Structural MRI: 3D MPRAGE T1-weighted, TR=2400 ms, TE=2.14 ms, TI=1000ms, flip angle=8°, FOV=224×224, voxel size=0.7mm isotropic. Diffusion-weighted MRI: spin-echo EPI, TR=5520ms, TE=89.5ms, flip angle=78°, FOV=208×180, 3 shells of b=1000, 2000, 3000smm−2 with 90 directions plus 6 b=0 acquisitions. Two sessions of 15min resting-state fMRI: gradient-echo EPI, TR=720 ms, TE=33.1ms, flip angle=52°, FOV=208×180, voxel size=2mm isotropic.

Area of acquisition: Whole-brain

Diffusion MRI: ☒ Used ☐ Not used

Parameters: For LAU, 128 diffusion-weighted volumes and a single b0 volume, maximum b-value 8,000 s/mm2, 2.2x2.2x3.0 mm voxel size
For HCP, 3 shells of b=1000, 2000, 3000 s/mm2 with 90 directions plus 6 b=0 acquisitions.

Preprocessing

Preprocessing software: For LAU, initial signal processing of all MPRAGE, DSI, and rs-fMRI data was performed using the Connectome Mapper pipeline (Daducci et al., 2012). Gray and white matter were segmented from the MPRAGE volume using freesurfer (Desikan et al., 2006). For HCP, MATLAB and toolboxes SPM8, DPARSF

Normalization: For LAU, the diffusion acquisition was used as the reference space and the tissue masks have been registered to the b0 volume using appropriate registration methods (Daducci et al., 2012). For HCP, data have been aligned across modalities and across subjects to Montreal Neurological Institute (MNI) standard space using appropriate volume-based and surface-based registration methods (see HCP documentation for details).

Normalization template: For LAU, data was analyzed after registering the T1-weighted image to the b0 volume, i.e. the volume acquired in absence of diffusion sensitising gradients. For HCP, data was analyzed after alignment to the MNI template.

Noise and artifact removal: For LAU, fMRI volumes were corrected for physiological variables, including regression of white matter, cerebrospinal fluid, as well as motion (three translations and three rotations, estimated by rigid body co-registration).
For HCP, voxel fMRI time courses were detrended and nuisance variables were regressed out (6 head motion parameters, average cerebrospinal fluid, and white matter signal).

Volume censoring: No censoring

Statistical modeling & inference

Model type and settings	General linear model performed for nuisance regression. Voxelwise timecourses are considered for functional connectivity analysis. NO effect tested.
Effect(s) tested	None, non relevant.

Specify type of analysis: ☐ Whole brain ☒ ROI-based ☐ Both

Anatomical location(s)	For LAU, gray matter was parcellated into 68 cortical nodes according to the Desikan–Killiany atlas (Desikan et al., 2006). These regions of interest were then further divided into 4 additional, increasingly finer-grained resolutions, comprising 114, 219, 448, and 1,000 approximately equally sized nodes (Cammoun et al., 2012).<br>For HCP, Glasser's multimodal cortical atlas (Glasser et al., 2016) converted to volume was used to parcellate the cortex into N=360 regions of interest.
Statistic type for inference (See Eklund et al. 2016)	None, non relevant.
Correction	None, non relevant.

Models & analysis

n/a	Involved in the study
☐	☒ Functional and/or effective connectivity
☐	☒ Graph analysis
☐	☒ Multivariate modeling or predictive analysis

Functional and/or effective connectivity	Pearson's correlation
Graph analysis	We built weighted structural connectivity graphs and estimated the connectivity as the density of fibers connecting two regions. A group-level connectome was obtained by averaging all subjects' structural matrices.
Multivariate modeling and predictive analysis	We utilized multilinear regression model and LASSO regression to perform regional structure-function prediction. The predictors are Laplacian eigenmodes of the structural connectome. The performance of structure-function prediction is evaluated as the Pearson correlation between predicted and empirical functional connectivity profiles of brain regions.
